# Natural-Killer-Derived Extracellular Vesicles: Immune Sensors and Interactors

**DOI:** 10.3389/fimmu.2020.00262

**Published:** 2020-03-13

**Authors:** Cristina Federici, Eriomina Shahaj, Serena Cecchetti, Serena Camerini, Marialuisa Casella, Elisabetta Iessi, Chiara Camisaschi, Giovanni Paolino, Stefano Calvieri, Simona Ferro, Agata Cova, Paola Squarcina, Lucia Bertuccini, Francesca Iosi, Veronica Huber, Luana Lugini

**Affiliations:** ^1^Department of Oncology and Molecular Medicine, Istituto Superiore di Sanità, Rome, Italy; ^2^Unit of Immunotherapy of Human Tumors, Fondazione IRCCS Istituto Nazionale dei Tumori, Milan, Italy; ^3^Core Facilities, Istituto Superiore di Sanità, Rome, Italy; ^4^Center for Gender-Specific Medicine, Istituto Superiore di Sanità, Rome, Italy; ^5^Dermatologic Clinic, La Sapienza University of Rome, Rome, Italy

**Keywords:** natural killer cells, microvesicles, exosomes, extracellular vesicles, immunosurveillance, healthy donors, melanoma patients

## Abstract

Natural killer (NK) cells contribute to immunosurveillance and first-line defense in the control of tumor growth and metastasis diffusion. NK-cell-derived extracellular vesicles (NKEVs) are constitutively secreted and biologically active. They reflect the protein and genetic repertoire of originating cells, and exert antitumor activity *in vitro* and *in vivo*. Cancer can compromise NK cell functions, a status potentially reflected by their extracellular vesicles. Hence, NKEVs could, on the one hand, contribute to improve cancer therapy by interacting with tumor and/or immune cells and on the other hand, sense the actual NK cell status in cancer patients. Here, we investigated the composition of healthy donors' NKEVs, including NK microvesicles and exosomes, and their interaction with uncompromised cells of the immune system. To sense the systemic NK cell status in cancer patients, we developed an immune enzymatic test (NKExoELISA) that measures plasma NK-cell-derived exosomes, captured as tsg101^+^CD56^+^ nanovesicles. NKEV mass spectrometry and cytokine analysis showed the expression of NK cell markers, i.e., NKG2D and CD94, perforin, granzymes, CD40L, and other molecules involved in cytotoxicity, homing, cell adhesion, and immune activation, together with EV markers tsg101, CD81, CD63, and CD9 in both NK-derived exosomes and microvesicles. Data are available via Proteome Xchange with identifier PXD014894. Immunomodulation studies revealed that NKEVs displayed main stimulatory functions in peripheral blood mononuclear cells (PBMCs), inducing the expression of human leukocyte antigen DR isotype (HLA-DR) and costimulatory molecules on monocytes and CD25 expression on T cells, which was maintained in the presence of lipopolysaccharide (LPS) and interleukin (IL)-10/transforming growth factor beta (TGFβ), respectively. Furthermore, NKEVs increased the CD56^+^ NK cell fraction, suggesting that effects mediated by NKEVs might be potentially exploited in support of cancer therapy. The measurement of circulating NK exosomes in the plasma of melanoma patients and healthy donors evidenced lower levels of tsg101^+^CD56^+^ exosomes in patients with respect to donors. Likewise, we detected lower frequencies of NK cells in PBMCs of these patients. These data highlight the potential of NKExoELISA to sense alterations of the NK cell immune status.

## Introduction

Natural killer (NK) cells belong to the innate immunity and represent the first-line defense of the immune system in the control of pathogens, tumor growth, and metastasis diffusion. NK cells constitute a population of large, granular lymphocytes that are located in the blood where they comprise 10–15% of lymphocytes, in lymphoid organs, i.e., thymus and spleen, and in non-lymphoid organs, such as the liver and uterus, as well as in tissues, i.e., skin (1, 2). NK cells are recognized as the most effective immune cells involved in immunosurveillance, as they can clear infected or transformed cells without the need for priming and are not restricted by the target cell's expression of major histocompatibility complex (MHC) molecules (3). Human peripheral blood NK cells can be identified by their expression of CD56 in the CD3^neg^ lymphocyte gate. They can be further divided into CD56^bright^CD16^+^ NK cells, which produce interferon gamma (IFNγ) and represent 10% of NK cell population, and into cytotoxic CD56^dim^CD16^bright^ NK cells, which represent 90% of the NK cell population under physiological conditions (4). NK cells constitutively express a lytic machinery able to kill target cells independently from any previous activation and can be induced to migrate toward inflammation sites and infected or transformed cells, by different chemoattractants (5). Previous studies have shown that a low activity of NK cells in peripheral blood is associated with increased cancer risk (6). A role of NK cells in tumor immunosurveillance has also been reported in models of spontaneous and induced tumors (7). In cancer patients, NK cells exhibit profound defects in the ability to degranulate (8). The therapeutic use of NK cells has been explored over the years, but the results have not been very encouraging. Considerable efforts are being made to achieve better activation, proliferation, and viability of NK cells, to be used in cancer immunotherapy (9).

Like other innate immune cells, NK cells communicate with dendritic cells and/or T and B cells and regulate innate and adaptive immune responses. NK cells do not act via antigen-specific mechanism but their activity is mediated by alternative receptors. The resulting inhibitory or activating signal depends on the ligand-expressing cells (10). This cell-to-cell communication is supported by the release of a broad range of soluble mediators, including cyto- and chemokines (11). Cells can also release soluble factors via encapsulation in extracellular vesicles (EVs) (12, 13). These nanometer-sized vesicles mainly comprise microvesicles (MV), stemming from the cell membrane with a diameter ranging from 150–1,000 nm and exosomes deriving from the endosomal compartment of the cells with a size ranging from 30 to 150 nm and apoptotic bodies, characterized by a dimension ranging from 1,000 to 5,000 nm (14). Among the different EV types, exosomes have captured the main interest from the scientific community for many years. Exosomes are important mediators for cell-to-cell communication in physiological (15–17) and pathological conditions such as cancer, where they play a major role in immune responses, cancer progression, and metastasis (18). Preclinical studies have shown that immune cells can release EVs with both stimulatory and tolerogenic properties into the extracellular microenvironment, a finding that supports their future application in cancer therapies (16, 19, 20). Moreover, there is a growing interest in the use of EVs, especially those deriving from cancer, as biomarkers, thanks to their simple detection in biological fluids, as recently shown for PD-L1^+^ exosomes in melanoma patients' plasma (21).

We were the first to report that NK cells release exosomes (NKExo), independently from the cells' activation status. NKExo express typical NK and EV markers together with killer proteins and are endowed with cytotoxic activity against tumor cells (22). In the years following their discovery, NKExo attracted major interest, and their antitumor activity was confirmed *in vitro* and *in vivo*, expanding the knowledge about these vesicles (10). Their therapeutic effect was also studied using NK cell lines as exosome producers to facilitate cell expansion for vesicle retrieval. Exosomes from the NK-92MI cell line showed remarkable cytolytic activity against melanoma (23) and glioblastoma (24), while EVs from *in vitro* expanded NK cells cocultured with K562.mbIL21 cells displayed antitumor activities against acute lymphoblastic leukemia and breast carcinoma (25). Apart from a consistent equipment of molecules aimed at target killing, NKEVs were also found to contain miR-186, a tumor suppressor micro-RNA that contributes to their cytostatic effects in neuroblastoma and prevents the inhibitory effects on NK cells of transforming growth factor beta (TGFβ) (26).

Here, we investigated the morphology and proteome of NK-cell-derived microvesicles (NKMV) and NKExo produced by *ex vivo* expanded NK cells from healthy donors and their effects on peripheral blood mononuclear cells (PBMCs) of healthy donors to uncover potential stimulatory activity on T cells, monocytes, and NK cells. Experiments recapitulating an immunosuppressed condition were performed in the presence of TGFβ/interleukin (IL)-10, tolerogenic conditioning of monocytes with lipopolysaccharide (LPS). In addition, we developed a method, the NKExoELISA, to sense alterations at EV level that could inform about the systemic NK cell immune status of cancer patients. Taken together, our data suggest that NKEVs could cover a promising role in the support of NK-mediated immunosurveillance to sustain cancer therapies, at the same time representing a sensor for systemic NK cell alterations.

## Materials and Methods

### NK Cell Expansion and PBMC Isolation

Blood of 20 healthy donors and 20 melanoma patients was provided by Centro Trasfusionale Universitario and Clinica Dermatologica of Azienda Policlinico Umberto I, University Sapienza, Rome, Italy. The study was approved by the ethical committee of Azienda Policlinico Umberto I, and subjects gave written informed consent to participate. Human PBMC were isolated with Ficoll-Histopaque 1077 gradient (Sigma-Aldrich, St. Louis, MO, United States). *Ex vivo* expanded human NK cells were obtained as previously described (22). Briefly, PBMCs from buffy coats were cocultured with cobalt-irradiated B lymphoblastoid Roswell Park Memorial Institute (RPMI) 8866 cells. On day 7, cells were incubated with human rIL-2 (100 U/ml; Hoffman-La Roche, Nutley, NJ, United States) for 3 days. The resulting NK cell population was >80% CD56^+^, CD3^−^, and CD14^−^ as assessed by flow cytometry analyses (cell viability, >90%). Using this culture method, an average of 30–40-fold increase in activated NK cell number was obtained. The supernatant of NK cell culture was properly frozen at −80°C for NKEVs isolation.

### Isolation of NKEVs

The culture supernatants of e*x vivo* expanded human NK cells were subjected to differential centrifugation as previously described (22). Briefly, conditioned cell culture medium was centrifuged for 5 min at 300 × *g* and 20 min at 1,200 × *g* to remove cells and debris; NKMVs were pelleted for 30 min at 10,000 × *g* and washed in phosphate-buffered saline (PBS), while NKExo were collected by ultracentrifugation at 100,000 × *g* for 90 min at 10°C using a Sorvall WX Ultra Series centrifuge in an F50L-2461.5 rotor (Thermo Scientific, Germany). The resulting pellet was washed in PBS and again ultracentrifuged at 100,000 × *g* for 60 min. MV or/and Exo was resuspended in PBS and RPMI 1640 medium or dissolved in lysis buffer for further analyses. To obtain plasma-derived exosomes, the plasma was centrifuged for 30 min at 500 × *g* and 45 min at 12,000 × *g* to collect microvesicles, filtered through a 0.22-μm filter (Sartorius, Germany), and ultracentrifuged for 2 h at 110,000 × *g* at 10°C to collect exosomes. The resulting pellet was washed in PBS, ultracentrifuged at 110,000 × *g* for 90 min, and properly preserved for subsequent analyses.

### Nanoparticle Tracking Analysis

The number and size of the isolated NK-derived EVs were assessed by nanoparticle tracking analysis (NTA) (NanoSight Model NS300, Malvern Instruments, NanoSight Ltd., Salisbury, United Kingdom). The parameters for NTA capture setting were as follows: camera type (sCMOS), Laser type Blue488, capture level 15, threshold 5, slider gain (366), and capture duration (60 s). Five videos of typically 60 s duration were taken. Data were analyzed by NTA 3.0 software (Malvern Instruments), which was optimized to first identify and then track each particle on a frame-by-frame basis.

### Microscopy Analysis

#### Phase Contrast Microscopy

Images were acquired with a Nikon Eclipse T100 inverted microscope (Nikon Instruments Inc., Melville, NY, United States) equipped with an LWD 10X 0.40 N.A. phase contrast objective, a Nikon DS-Fi1 color camera, and the NIS-Elements F v3.0 software (Nikon Instruments Inc.).

#### Electron Microscopy

For scanning electron microscopy (SEM), purified exosomes and microvesicles from NK cell supernatants (>10^9^/ml) were suspended in PBS and left to adhere to polylisine-treated round glass coverslips (Ø10 mm). Samples were fixed with 2.5% glutaraldehyde in 0.1 M Na-cacodylate buffer and processed for SEM as previously described with slight modifications (27). Briefly, samples were postfixed with 1% OsO_4_ in 0.1 M sodium cacodylate buffer and were dehydrated through a graded series of ethanol solutions (from 30 to 100%). Then, absolute ethanol was gradually substituted by a 1:1 solution of hexamethyldisilazane (HMDS)/absolute ethanol and successively by pure HMDS. The final drying process was concluded, removing completely the HMDS and leaving to evaporate all the liquid phase on air and in a desiccator. Dried samples were mounted on stubs, coated with gold (10 nm), and analyzed in an FE-SEM Quanta Inspect F (FEI-Thermo Fisher Scientific). For transmission electron microscopy (TEM), for negative staining, a pellet of extracellular vesicle purified from NK cell supernatants was suspended in PBS and were deposited on carbon-coated grids for electron microscopy. Phosphotungstic acid 2% was added on grids to give sample contrast. Samples were air dried and observed with a PHILIPS EM208S transmission electron microscope (FEI-Thermo Fisher) (28).

#### Immunoelectron Microscopy

Either purified exosomes and microvesicles from healthy donor NK cells were suspended in PBS and adsorbed on carbon-coated grids for electron microscopy, accordingly to Thery group's protocol (28) with slight modifications. Samples were air dried, and all the successive passages were carried out by floating the grids on drops of different solutions. First, the grids were floated on anti-CD81 (mAb B11, Santa Cruz Biotechnology, Heidelberg, Germany) or anti-CD56 (mAb 123C3, Santa Cruz) monoclonal antibodies, rinsed on buffer and incubated on 10 nm gold-conjugated goat antimouse immunoglobulin G (IgG) serum (Sigma-Aldrich, St. Louis, MO). Then, samples were rinsed and incubated on 2% paraformaldehyde. Finally, after rinsing on water, the samples were contrasted with 2% ammonium molybdate (“positive-negative” contrast) and dried with a filter paper. Samples were observed with a PHILIPS EM208S transmission electron microscope (FEI-Thermo Fisher).

### Proteomics Analysis

Proteins obtained from three pools of total NK cell extracts, NKExo, and NKMV derived from three healthy donors each were loaded (15 μg for each sample), separated on a 1D-gel NuPAGE 4–12% (Novex, Invitrogen, CA, United States), and stained with Coomassie blue (Colloidal Blue Staining kit, Invitrogen). Each lane was cut in six contiguous slices that were treated with dithiothreitol (DTT) and iodoaceatamide and finally digested with trypsin (Promega Corporation, WI, United States) as previously described (29). Peptides were analyzed by liquid chromatography–MS/MS (LC-MS/MS) on an Orbitrap Fusion Tribrid mass spectrometer (Thermo Fisher Scientific, CA, United States) equipped with an Ultimate 3000 UHPLC (Dionex, Thermo Fisher Scientific). Peptides were desalted on a trap column (Acclaim PepMap 100 C18, Thermo Fisher Scientific) and then separated on a 20-cm-long silica capillary (Silica Tips FS 360-75-8, New Objective, MA, United States), packed in-house with a C18, 5 μm, 100 Å resin (Michrom BioResources, CA, United States).

The analytical separation was run for 91 min using a gradient of buffer A (95% water, 5% acetonitrile, and 0.1% formic acid) and buffer B (95% acetonitrile, 5% water, and 0.1% formic acid).

The gradient was run as follows: buffer A was fixed at 5% for the first 5 min, then was linearly increased to 30% in 60 min and subsequently to 80% in 10 min, then remained at 80% for 5 min, and finally decreased at 5% for a 10-min long column re-equilibration step.

Full-scan MS data were acquired in the 350–1,550 *m*/*z* range in the Orbitrap at 60 k resolution. Data-dependent acquisition was performed using top-speed mode (3 s long maximum total cycle): the most intense precursors were selected through a monoisotopic precursor selection (MIPS) filter and with charge >1, quadrupole isolated and fragmented by higher-energy collision dissociation (HCD) (32 collision energy). Fragment ions were analyzed in the ion trap with rapid scan rate. The automatic gain control (AGC) target value was set to 4e5 (FT) for MS1 and 2e3 (IT) for MS2. Maximum injection times of 50 and 100 ms were used for MS1 and MS2, respectively. Raw data were analyzed by Proteome Discoverer 2.3 (Thermo Fisher Scientific) using the human database from UniProtKB/Swiss-Prot database (Release 25 October 2017; 42253 sequences). Spectral matches were filtered using Percolator node, based on *q* values, with 1% false discovery rate (FDR). Only master proteins were taken into account and considered identified with at least two peptides with specific trypsin cleavages with two miscleavages admitted. Cysteine carbamydomethylation was set as static modification, while methionine oxidation and *N*-acetylation on protein terminus were set as variable modifications.

Quantification was based on precursor intensity using only unique peptides and normalizing abundances on total peptide amount. The sample group abundance was calculated as the median abundance of biological replicates. Protein abundance ratios for each couple of sample groups NKExo/NKTotExtr and NKMV/NKTotExtr were calculated as a pairwise protein ratio: no imputation was applied, and *t*-test and *P*-values corrected for multiple testing using the Benjamini–Hochberg procedure were adopted to evaluate significant differences in protein abundances. Proteins with an abundance ratio >1.5 and *P* < 0.05 were considered differentially expressed. Proteome Discoverer was also applied to get information about correlation among the data: normalized abundances were used for principal component analysis (PCA), and hierarchical cluster analysis of the grouped abundances was obtained.

All the identified proteins were analyzed to recognize species unique or shared among sample groups (http://bioinformatics.psb.ugent.be/webtools/Venn/) and compared with Exocarta (http://www.exocarta.org/) (30) and Vesiclepedia (http://www.microvesicles.org) (31) database. Overexpressed proteins were evaluated for protein–protein interaction analysis using Search Tool for the Retrieval of Interacting Genes/Proteins (STRING) (https://string-db.org/) considering high confidence data with minimum required interaction score of 0.7; cellular component and Reactome pathway enrichment were also evaluated with FDR < 1E−3 (32). Gene Ontology (GO) terms relative to cellular component or biological process were also analyzed separately in the proteins enriched in NKExo (proteins overexpressed in NKExo and those identified in NKExo or NKExo plus NKMV) and proteins enriched in NKMV (proteins overexpressed in NKMV and those identified in NKMV or NKMV plus NKExo) using DAVID version 6.8 (https://david.ncifcrf.gov/), taking into account GO terms with Benjamini *P* < 1E−3 and FDR < 1E−3 (33) and considering all the proteins identified in the NK total cell extract as our background. All the mass spectrometry proteomics data have been deposited to the ProteomeXchange Consortium via the PRIDE (34) partner repository with the dataset identifier PXD014894.

### Cytokine Bead Array

NK-derived EVs (15 μg NKMV and 30 μg NKExo) and supernatants of coculture experiments were assayed for the presence of cyto-chemokines by cytokine bead array (CBA, Becton Dickinson, San Diego, CA, United States) according to manufacturer's instructions. Samples were acquired with a FACSCalibur flow cytometer (Becton Dickinson), and data were analyzed by the FCAP Array software (Becton Dickinson).

### Coculture Experiments

All experiments were performed using RPMI 1640 medium (Lonza Verviers, Belgium), supplemented with 10% bovine EV-depleted fetal bovine serum (FBS), 100 U/ml penicillin, 100 mg/ml streptomycin (Lonza), and 2 mmol/L glutamine in a 5% CO_2_ environment at 37°C. All cell lines were negative for mycoplasma contamination, as routinely tested by a PCR mycoplasma detection kit (Venor GeM; Minerva Biolabs, Berlin, Germany). Before coculture experiments with CD3/CD28 stimulation, PBMCs were labeled with carboxyfluorescein diacetate succimidyl ester (CFSE; Molecular Probes, Invitrogen Technologies, MA, United States). To study the activity of NKEVs on monocytes, 10^5^ PBMCs or purified CD14^+^ cells from healthy donors were incubated with 15 μg isolated NKMV or 30 μg NKExo for 24 h. PBMC experiments included conditions in the presence or absence of LPS (250 ng/ml). Effects of NKMV or NKExo were also investigated on CFSE-labeled PBMCs or CD14-depleted PBMCs stimulated or not for 72 h with anti-CD3 (plate bound, 1 μg/ml; OKT3 Orthoclone, Janssen Biotech, Inc. PA, United States) and anti-CD28 (1 μg/ml; Becton Dickinson, NJ, United States). To mimic an immunosuppressive environment, experiments with CFSE-labeled PBMC were also performed in the presence of recombinant IL-10 (10 ng/ml; Peprotech, NJ, United States) and recombinant TGFβ (10 ng/ml; Peprotech). At the end of the incubation, cells were harvested and incubated with fluorochrome-conjugated Abs to allow phenotyping of monocytes, NK cells, and T cells. Supernatants were collected from coculture experiments for cyto-chemokine detection by cytokine bead array. To test the stimulatory activity of NKEV-conditioned monocytes on activated T cells, CD14^+^ cells, isolated from PBMCs with anti-CD14^+^ beads (MACS, Miltenyi Biotec, Germany) according to the manufacturer's instructions, were cultured in the presence of NKEVs (15 μg NKMVs or 30 μg NKExo) for 24 h. After a washing step to eliminate excess NKEVs, monocytes were added to the autologous CD14-negative fraction, previously labeled with CFSE, in the presence of T-cell stimuli anti-CD3/antiCD28, as described above. At the end of the incubation (72 h), cells were harvested and evaluated for proliferation and CD25 expression by flow cytometry.

### Flow Cytometry

Acquisitions were performed using a Beckman Coulter Cytoflex flow cytometer, and data were analyzed by the Kaluza software (Beckman Coulter, Milan, Italy). Cytoflex Daily QC Fluorospheres (Beckman Coulter) were used to calibrate the flow cytometer. The following monoclonal fluorochrome-conjugated antibodies were used for cell labeling of CD14^+^ cells from NKEV-monocyte cultures and PBMCs harvested from LPS-stimulated NKEV-PBMC cocultures: CD3-KO (Beckman Coulter), CD14-Alexa 750 (Beckman Coulter), CD16-BV650 (Biolegend), CD80-86-PE (Becton Dickinson), and HLA-DR-APC (Beckman Coulter).

The following antibodies were used for cell labeling of peripheral blood lymphocytes (PBL), NK cells and PBMCs harvested from CD3/CD28-stimulated or unstimulated NKEV-PBMC cocultures, in the presence or absence of TGFβ/IL-10: CFSE (CellTrace, Thermo Fisher Scientific), CD3-ECD (Beckman Coulter), CD4-Alexa700 (Beckman Coulter), CD8-BV605 (Biolegend), CD56-BV510 (Becton Dickinson), CD16-BV650 (Biolegend), PD-1-PC7 (Beckman Coulter), CD25-PercpCy5.5 (Becton Dickinson), HLA-DR-APC (Beckman Coulter), and CD14-Alexa750 (Beckman Coulter).

Samples were incubated with Fc blocking reagent (Miltenyi Biotec, Germany) for 10 min at room temperature before the addition of monoclonal antibodies for 40 min at 4°C. Thereafter, samples were washed and fixed.

### NKExoELISA

To measure NK exosomes isolated from the plasma of healthy donors and melanoma patients, a homemade specific immune-enzymatic test, the NKExoELISA, was conducted. Briefly, 96-well plates (Nunc, Milan, Italy) were coated with 4 μg/ml rabbit polyclonal anti-tsg101 antibody (ab70974, Abcam, Cambridge, United Kingdom) and incubated overnight at 4°C. After 1× PBS washes, a blocking solution [0.5% bovine serum albumin (BSA) in 1× PBS] was added at room temperature for 1 h. Following PBS washes, nanovesicles isolated by differential centrifugation of 1.0 ml of plasma were incubated overnight at 37°C. After PBS washes, 4 μg/ml of monoclonal anti-CD9 (ab2215, Abcam) or monoclonal anti-CD56 (123C3, Santa Cruz) was added for 1 h at 37°C. After three washes with PBS, the plate was incubated with horseradish peroxidase (HRP)-conjugated antimouse antibody (Pierce, Milan, Italy) for 1 h at room temperature, and the reaction was developed with Blue POD for 15 min (Roche Applied Science, Milan, Italy) and blocked with 1 N H_2_SO_4_ stop solution, and optical densities were recorded at 450 nm.

### Statistical Analysis

Data are expressed as mean ± SEM or mean ± SD, as indicated, using GraphPad Prism, and *P*-values of 0.05 or less were considered to be significant. The statistical analysis was performed by paired and unpaired Student's *t*-test, as indicated.

## Results

### Characterization of NKEVs: Microvesicles and Exosomes

**Figure 1:**
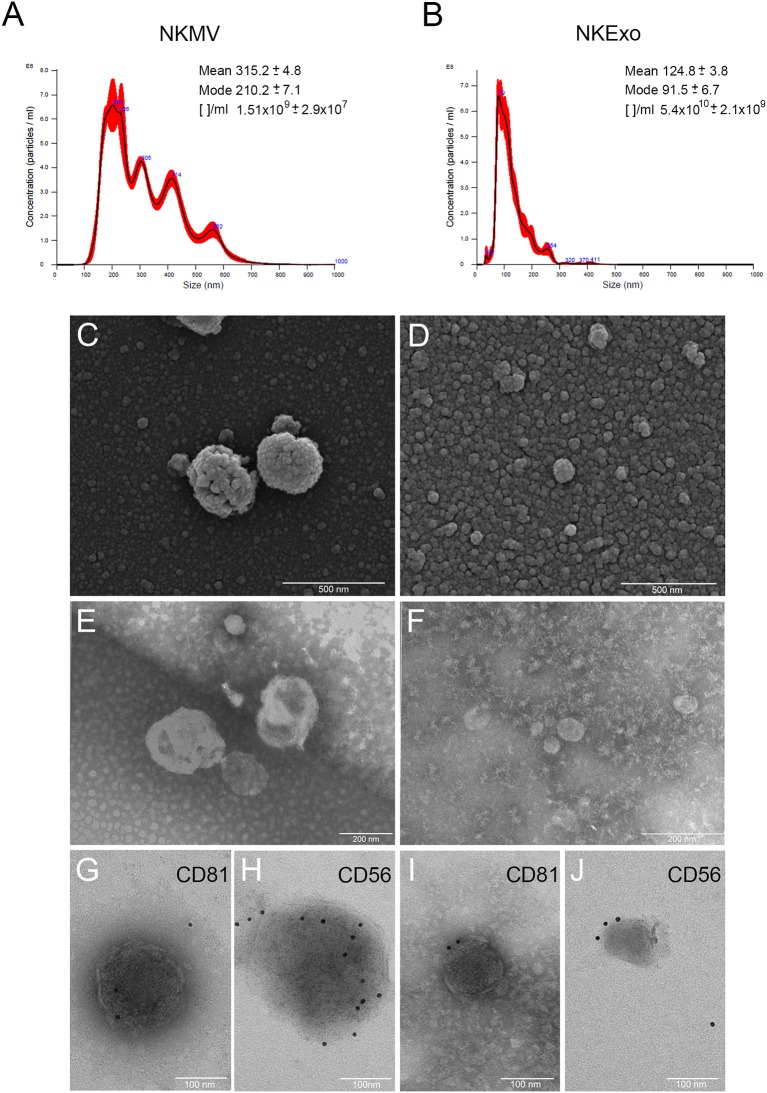
Morphological characterization of natural killer cell-derived extracellular vesicles (NKEVs). **(A,B)** Nanoparticle tracking analysis (NTA) analysis of NK-cell-derived microvesicle (NKMV) and NKExo. Representative spectra are shown, and mean, mode, and particles number/ml ([]/ml) are reported. **(C,D)** Scanning electron microscopy analysis of NKMV **(C)** and NKExo **(D)**. Bars, 500 nm. **(E,F)** Transmission electron microscopy analysis of NKMV **(E)** and NKExo **(F)**. Bars, 200 nm. **(G–J)** Immunoelectron microscopy combined with positive/negative contrast method of NKMV **(G,H)** and NKExo **(I,J)** revealed the presence of CD81 **(G,I)** and CD56 **(H,J)**. Bars, 100 nm.

MV and Exo produced by *in vitro*-amplified NK cells from healthy donors were isolated by differential centrifugation and characterized. NTA revealed bigger dimensions and major heterogeneity of NKMV as compared to NKExo (315.2 vs. 124.8 nm mean value, respectively). NKExo appeared more concentrated than NKMV (5.4 × 10^10^ vs. 1.51 × 10^9^/ml, respectively) (Figures 1A,B). We calculated that a single NK cell produced 0.6 × 10^3^ MVs and 2.1 × 10^4^ Exo in our cell culture condition.

Scanning electron microscopy analysis (Figures 1C,D) showed an overview of NKMV and NKExo and confirmed a high density of both types of EVs in the 10,000 × *g* fraction (Figure 1C), while that one purified at 100,000 × *g* consisted prevalently of vesicles with dimensions smaller than 150 nm, which were accordant with an exosome population (Figure 1D). Transmission electron microscopy analysis confirmed the membranous nature of both kinds of EVs (Figures 1E,F) and revealed once again the quite exclusive presence of exosome-like vesicles in the 100,000 × *g* pellet (Figure 1F). Immunoelectron microscopy combined with positive/negative contrast method revealed the presence of CD81 EV marker (Figures 1G,I) and CD56 NK cell marker (Figures 1H,J) in both kinds of EVs.

### Proteomic Profiles of NKEVs

**Figure 2:**
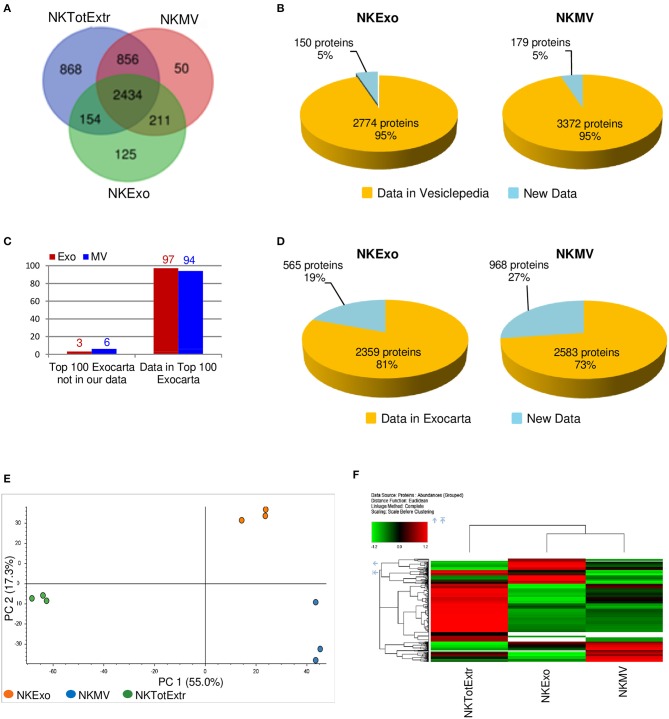
Proteomic analysis of proteins identified in natural killer (NK) cell total extract, NK-cell-derived microvesicle (NKMV) and NKExo. **(A)** Venn diagram of all identified proteins in NK cell total extract (TotExtr), NKMV, and NKExo. **(B)** Proteins identified in NKExo and NKMV samples compared with those present in Vesiclepedia database. **(C,D)** Proteins identified in NKExo (Exo) and NKMV (MV) samples compared with those present in the top 100 Exocarta **(C)** and in the whole Exocarta database **(D). (E)** Principal component analysis (PCA) of NKExo, NKMV, and TotExtr normalized protein abundances. **(F)** Hierarchical cluster analysis of the grouped abundances of NKExo, NKMV, and TotExtr scaled before clustering and using the euclidean function for the distance.

To characterize healthy donor circulating NKMV and NKExo, we performed a proteomic analysis by LC-MS/MS of a biological triplicate constituted by three pools, derived from three different healthy donors each, in parallel to their parental *ex vivo*-expanded NK total cell extracts. Relative protein abundances were similar in all samples (Figure S1), and a total of 4,698 proteins were identified (Figure 2A; Table S1). Overall, 3,830 proteins were comprehensively identified in NKExo and NKMV: some of these proteins were also detected in the total cell extracts, while some others were specifically identified in NKExo (125 proteins) or NKMV (50 proteins) or in both (211 proteins) preparations. The majority of NKExo proteins as well as most of the NKMV proteins were present in the Vesiclepedia (95% for both) or Exocarta (81 and 73%, respectively) databases (Figures 2B,D). In addition, 97 and 94% of the Top 100 proteins reported in the Exocarta database were also detectable in our preparations of NKExo and NKMV, respectively (Figure 2C), confirming a high enrichment of proteins previously identified in vesicles and/or exosomes. In particular, we have examined the relative amount of proteins expected to be enriched or not in the vesicles (both in NKExo and NKMV) according to categories 1–3 described by Lötvall and coworkers (35) (Figures S2–S6). As expected, we detected an enrichment of several proteins belonging to category 1, including tetraspanins (CD9, CD81, and CD82), integrins (integrin alpha-6, beta-3), flotilins (Flotilin-1 and Flotilin-2), and cell adhesion proteins (ICAM1 and VCAM1), and category 2 (Syntenin-1, ALIX, and Annexin A3) both in NKExo and NKMV. Conversely, proteins expected not to be enriched in EVs, defined as category 3, including endoplasmic reticulum associated proteins (ERAP1, ERMP1, ERP29, and Calnexin), mitochondrial proteins (mitochondrial 2-oxoglutarate/malate carrier protein, mitochondrial dicarboxylate carrier, mitochondrial glutamate carrier, and mitochondrial proton/calcium exchanger protein), and the protein Ago1 were enriched in the parental cell extracts with respect to their vesicles. This analysis showed that the protein characteristics of our NKEVs are in line with previous definitions of EVs (35).

Principal component analysis was carried out on proteins identified in each individual biological replicate for each sample to evaluate the similarity among the different data sets (Figure 2E): the first and second principal component (PC1 and PC2) together account for ~72% of the total sample variance and indicated low variance between biological replicates and a distinct separation among NKExo, NKMV, and cells. In particular, the predominant variance (55%) in the proteome content derived from differences between particles (NKMV and NKExo) and total cell extracts, while the second component (17%) evidenced the low level of similarity between NKMV and NKExo, as distinct from parental NK cell profiles. Differences in protein abundance profiles in NKMV, NKExo, and total cell extracts were compared using hierarchical clustering (Figure 2F): this relative quantitative analysis confirmed that the variance among the biological replicates was lower than that observed among sample groups (data not shown) and highlighted that NKExo and NKMV protein profiles shared a low level of similarity and were together highly different from that of total NK cell extract.

**Figure 3:**
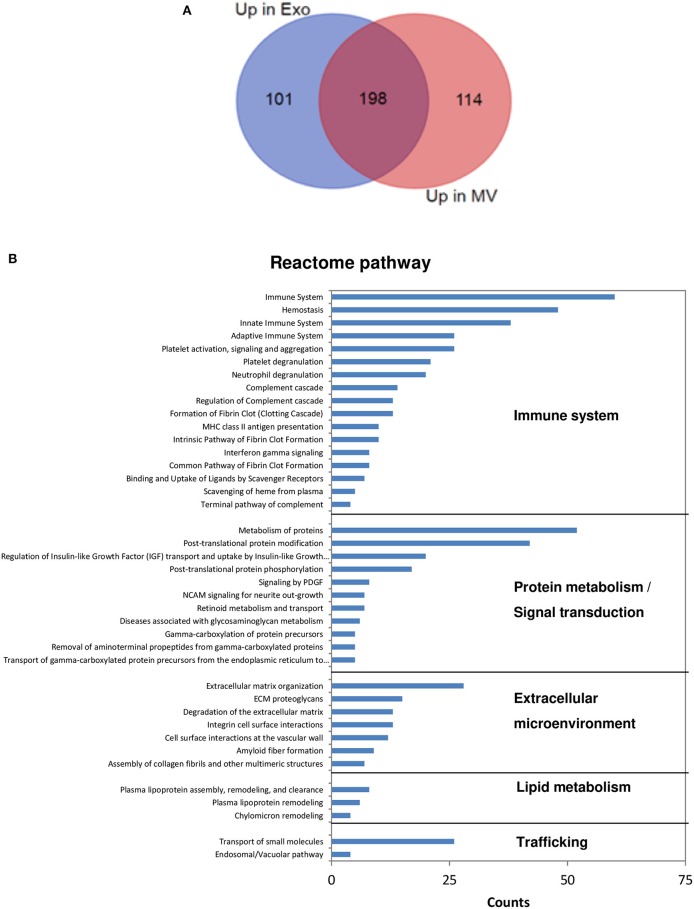
Analysis of proteins overexpressed in NKExo and/or NK-cell-derived microvesicle (NKMV). **(A)** Venn diagram displaying unique and shared overexpressed proteins in NKExo (Up in Exo) and NKMV (Up in MV). **(B)** Reactome pathway annotation of proteins overexpressed in NKExo and/or NKMV obtained by Search Tool for the Retrieval of Interacting Genes/Proteins (STRING). The number of proteins (counts) involved in each Gene Ontology (GO) term is displayed.

Differential protein expression among samples was examined by label-free approach: proteins with abundance ratio >1.5 and *P* < 0.05 were considered as significantly differentially expressed. A total number of 413 proteins were overexpressed in NKExo and/or NKMV in comparison with total cell extract, 48% of which was increased in both sets, while 24 and 28% were uniquely overexpressed in NKExo and NKMV, respectively (Figure 3A, Table S2). STRING analysis showed enrichment in protein–protein interactions (PPI enrichment, *P* < 1.0E−16) and in the EV cellular component (with 115 proteins belonging to extracellular region GO term 0005576 with FDR of 3.7E−28). Term enrichment in Reactome pathway knowledge revealed a relevant enrichment in proteins involved in the immune system followed by those implicated in protein metabolism and signal transduction, extracellular microenvironment, trafficking, and lipid metabolism (Figure 3B).

**Figure 4:**
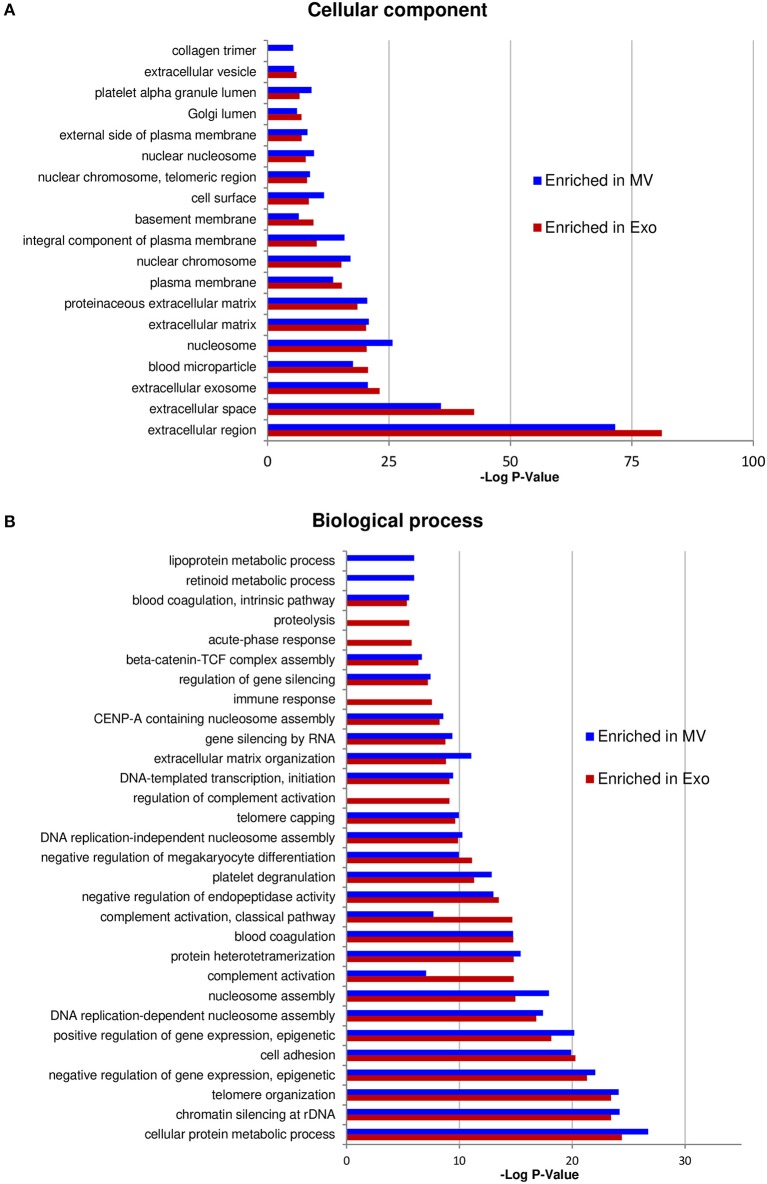
Analysis of proteins enriched in NKExo and/or NK-cell-derived microvesicles (NKMV). Gene Ontology (GO) annotation of proteins overexpressed in NKExo and identified in NKExo or NKExo plus NKMV (enriched in Exo) and proteins overexpressed in NKMV and identified in NKMV or in NKMV plus NKExo (enriched in MV). The significance [–log_10_(*P*-value)] for each GO term relative to cellular component **(A)** and biological process **(B)** is reported.

We then analyzed GO terms relative to cellular component and biological process separately in proteins enriched in NKExo (assembling proteins overexpressed in NKExo and those identified in NKExo and in NKExo plus NKMV) and proteins enriched in NKMV (assembling proteins overexpressed in NKMV and those identified in NKMV and in NKMV plus NKExo) (Figure 4). Cellular component terms were very similar between NKExo- and NKMV-enriched proteins, with the most important enrichment in extracellular region and extracellular exosome, as expected. NKExo and NKMV also shared several GO terms belonging to biological process annotation, but in some cases with different degrees of significance, as in the case of complement activation, immune response, and proteolysis, which resulted specifically significant among proteins enriched in NKExo. On the other side, retinoid and lipoprotein metabolic processes resulted in a significant unique enrichment in NKMV. These differences probably reflected a slightly different nature and function between NKExo and NKMV.

**Figure 5:**
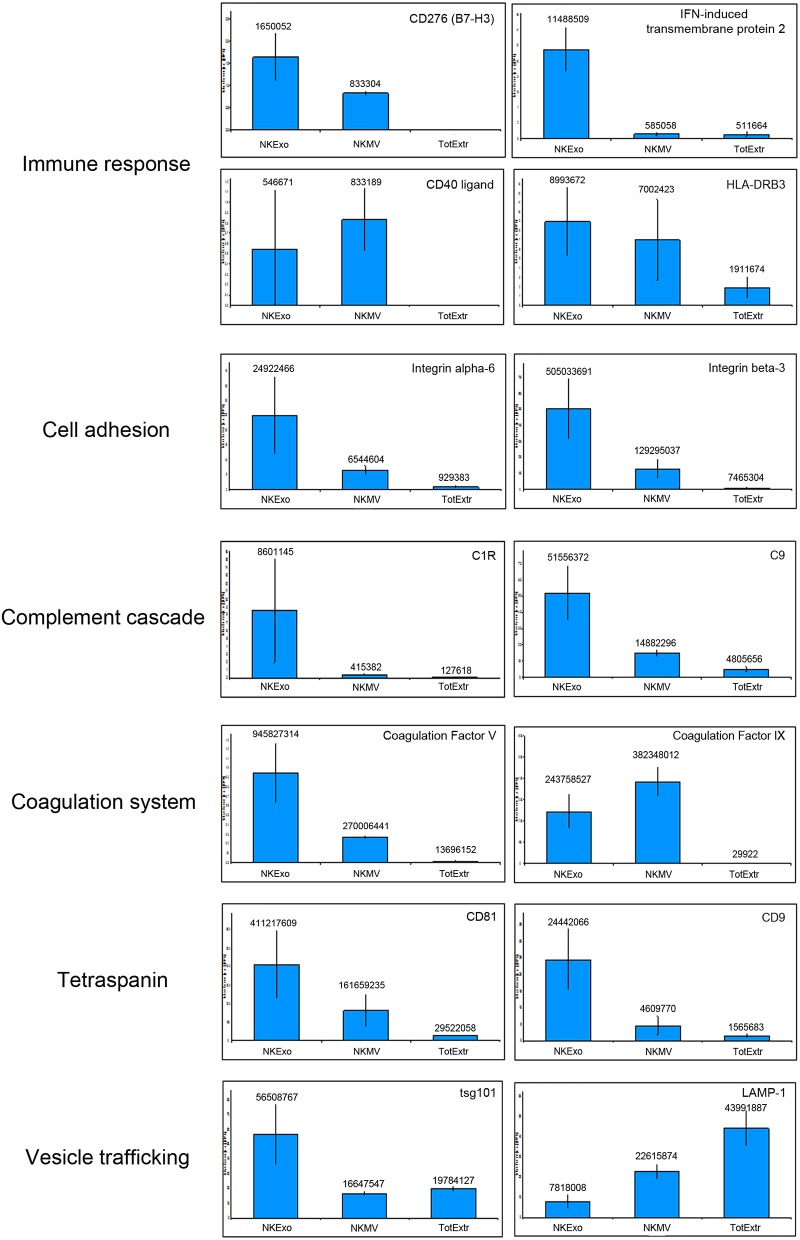
Protein abundances of specific proteins quantified by liquid chromatography–MS/MS (LC-MS/MS). Representative proteins grouped in the indicated categories are shown. Each panel shows the protein median abundance of the three replicates of NKExo, NK-cell-derived microvesicle (NKMV), NK total cell extract (TotExtr). The bars indicate the standard error for each sample group.

**Figure 6:**
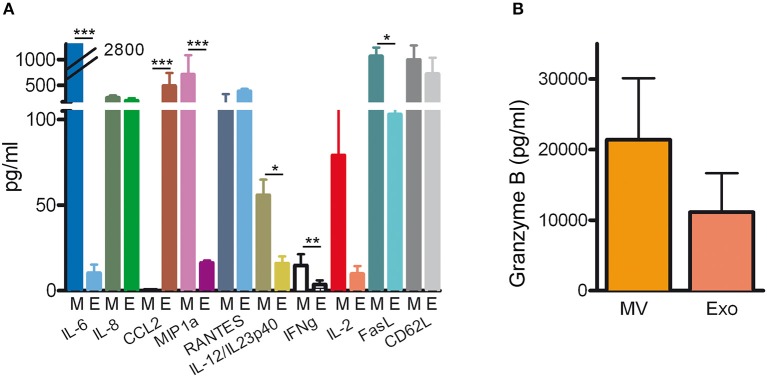
Cyto- and chemokine profiling natural killer cell-derived extracellular vesicles (NKEVs). **(A)** Cytokine bead array for the indicated soluble factors in NK-cell-derived microvesicle (NKMV) (M) and NKExo (E). **(B)** Granzyme B expression by cytometric bead array (CBA) assay in NKMV (MV) and NKExo (Exo). Results are shown for NKEVs produced by different healthy donors (*n* = 3). Statistical significance was achieved by paired *t*-test. Means ± SD are plotted, *p < 0.05; **p < 0.01; ***p < 0.001.

Among all the identified proteins in NKEVs, we selected 165 proteins playing key roles in the biology of NK cells and belonging to different clusters of cellular processes such as immune response, cell adhesion, complement activation, and blood coagulation (Table 1). As shown in Figure 5, a large amount of proteins were overexpressed by NKEVs compared to NK total extracts. These proteins belong to multiple categories with different biological roles, comprising cell adhesion, immune response, coagulation, and vesicle trafficking. Some proteins such as CD276, interferon-induced transmembrane protein 2, and tsg-101 were upregulated in NKExo as compared to that in NKMV. Other proteins, such as CD40 ligand, lysosomal-associated membrane protein 1 (LAMP-1), and coagulation factor IX were upregulated in NKMV, with respect to NKExo. These data suggest that even though NKMV and NKExo express the same proteins they might have different roles in the same pathways. Additional cyto- and chemokine profiling was performed by cytokine bead array (CBA) of NKMV (15 μg) and NKExo (30 μg) isolated from the same starting volume of NK cell culture supernatant. This was the condition we chose for coculture experiments based on our previous work with NK exosomes (22), where we used 30 μg of exosomes to obtain a biological effect. In the case of MV, the amount of protein that we recovered was much less compared to exosomes; thus, based on 30 μg NKExo, we used the maximum amount possible of MV, isolated from the same volume of supernatant as exosomes. CBA results confirmed that both types of vesicles carry remarkable amounts of different soluble factors. A similar distribution in MV and Exo could be measured for IL-8, regulated upon activation, normal T cell expressed and secreted (RANTES), and CD62L, while IL-6, IL-2, IFNγ, IL-12/IL23p40, FasL, and macrophage inflammatory protein 1α (MIP1α) appeared to concentrate in the MV and CCL2 in the Exo fraction. Considering both populations, we could detect higher concentrations of IL-6, FasL, CD62L, RANTES, MIP1α, and CCL2 with respect to IL-12/IL-23p40, IFNγ, and IL-2 (Figure 6A). Moreover, we could detect both in NKMV and NKExo a large amount of granzyme B, known to be a strong cytokine modulator as well as a potent cytotoxicity inducer (Figure 6B).

**Table 1:** Selected proteins identified in natural killer cell-derived extracellular vesicles (NKEVs).

**Category**	**Accession**	**Description**	**Entrez Gene ID**	**Up in MV^a^**	**Up in Exo^b^**
Cell adhesion	P22303-1	Acetylcholinesterase	43	Up	Up
	Q9UHX3	Adhesion G protein-coupled receptor E2	30817	Up	Up
	Q9NVD7	Alpha-parvin	55742	Up	Up
	Q9HBI1-1	Beta-parvin	29780	Up	Up
	Q99828	Calcium and integrin-binding protein 1	10519	/	Up
	P49747	Cartilage oligomeric matrix protein	1311	Up	Up
	P48960-1	CD97 antigen	976	/	/
	P02452	Collagen alpha-1(I) chain	1277	/	/
	P20908	Collagen alpha-1(V) chain	1289	/	Up
	P12109	Collagen alpha-1(VI) chain	1291	Up	Up
	P39059	Collagen alpha-1(XV) chain	1306	Up	Up
	P12110	Collagen alpha-2(VI) chain	1292	Up	Up
	P12111	Collagen alpha-3(VI) chain	1293	Up	Up
	A6NMZ7-1	Collagen alpha-6(VI) chain	131873	Up	Up
	P29279-1	Connective tissue growth factor	1490	Up	Up
	Q12860	Contactin-1	1272	/	Up
	Q14574-1	Desmocollin-3	1825	/	/
	O43854-1	EGF-like repeat and discoidin I-like domain-containing protein 3	10085	/	/
	P17813	Endoglin	2022	Up	Up
	P02751	Fibronectin	2335	Up	Up
	Q14520-1	Hyaluronan-binding protein 2	3026	Up	Up
	Q13308-1	Inactive tyrosine-protein kinase 7	5754	/	Up
	P35858	Insulin-like growth factor-binding protein complex acid labile subunit	3483	/	Up
	P56199	Integrin alpha-1	3672	/	/
	P17301	Integrin alpha-2	3673	/	/
	P13612	Integrin alpha-4	3676	/	/
	P08648	Integrin alpha-5	3678	/	/
	P23229-1	Integrin alpha-6	3655	Up	Up
	P08514-1	Integrin alpha-IIb	3674	/	/
	P20701-1	Integrin alpha-L	3683	/	/
	P11215-1	Integrin alpha-M	3684	/	/
	P06756	Integrin alpha-V	3685	/	/
	P20702	Integrin alpha-X	3687	/	/
	P05556-1	Integrin beta-1	3688	/	/
	P05107	Integrin beta-2	3689	/	/
	P05106	Integrin beta-3	3690	Up	Up
	P26010-1	Integrin beta-7	3695	/	/
	Q08431	Lactadherin	4240	/	Up
	P07942	Laminin subunit beta-1	3912	/	/
	P55083	Microfibril-associated glycoprotein 4	4239	Up	Up
	P35580	Myosin-10	4628	Up	Up
	Q92859	Neogenin	4756	/	/
	Q14112-1	Nidogen-2	22795	/	Up
	Q15063-1	Periostin	10631	Up	Up
	P13224	Platelet glycoprotein Ib beta chain	2812	Up	Up
	P14770	Platelet glycoprotein IX	2815	Up	Up
	Q12884-1	Prolyl endopeptidase FAP	2191	Up	Up
	Q14517	Protocadherin Fat 1	2195	Up	/
	Q9UN70-1	Protocadherin gamma-C3	5098	Up	Up
	P16109	P-selectin	6403	Up	Up
	Q13332	Receptor-type tyrosine-protein phosphatase S	5802	/	Up
	P78509	Reelin	5649	/	Up
	P05026	Sodium/potassium-transporting ATPase subunit beta-1	481	Up	/
	P24821	Tenascin	3371	Up	/
	P22105	Tenascin-X	7148	Up	Up
	P16591-1	Tyrosine-protein kinase Fer	2241	/	/
	P19320-1	Vascular cell adhesion protein 1	7412	/	/
Cell adhesion/immune response	Q13740-1	CD166 antigen	214	/	/
	P24001	Interleukin-32	9235	/	Up
	P16671	Platelet glycoprotein 4 CD 36	948	/	/
	P07996	Thrombospondin-1	7057	Up	Up
	P35442	Thrombospondin-2	7058	/	/
	P49746	Thrombospondin-3	7059	/	/
	P35443	Thrombospondin-4	7060	Up	Up
	O43294-1	Transforming growth factor beta-1-induced transcript 1 protein	7041	Up	Up
	Q15582	Transforming growth factor-beta-induced protein ig-h3	7045	Up	Up
	P04004	Vitronectin	7448	Up	Up
	P14151	L-Selectin	6402	/	/
Immune response	P13727	Bone marrow proteoglycan	5553	/	/
	P32248	C–C chemokine receptor type 7	1236	Up	/
	Q5ZPR3	CD276 antigen	80381	Up	Up
	P29965	CD40 ligand	959	Up	Up
	P09326	CD48 antigen	962	/	/
	P32970	CD70 antigen	970	Up	/
	P49682-1	C–X–C chemokine receptor type 3	2833	/	/
	P61073	C–X–C chemokine receptor type 4	7852	/	/
	Q86WI1	Fibrocystin-L	93035	/	/
	Q9Y6W8-1	Inducible T-cell costimulator	29851	Up	/
	P13164	Interferon-induced transmembrane protein 1	8519	/	/
	Q01629	Interferon-induced transmembrane protein 2	10581	/	/
	Q01628	Interferon-induced transmembrane protein 3	10410	/	Up
	Q9NPH3	Interleukin-1 receptor accessory protein	3556	/	Up
	Q13478	Interleukin-18 receptor 1	8809	/	/
	P14784	Interleukin-2 receptor subunit beta	3560	/	/
	Q9BZW8-2	Isoform 2 of Natural killer cell receptor 2B4	51744	/	/
	P43627	Killer cell immunoglobulin-like receptor 2DL2	3803	/	/
	P43628-1	Killer cell immunoglobulin-like receptor 2DL3	3804	/	/
	O43561	Linker for activation of T-cells family member 1	27040	Up	Up
	Q9GZY6	Linker for activation of T-cells family member 2	7462	/	/
	O14931-1	Natural cytotoxicity triggering receptor 3	259197	/	/
	Q13241	Natural killer cells antigen CD94	3824	/	/
	P26715	NKG2-A/NKG2-B type II integral membrane protein	3821	/	/
	P26718-1	NKG2-D type II integral membrane protein	22914	**/**	**/**
	Q92626	Peroxidasin homolog	7837	**/**	**/**
	P42081	T-lymphocyte activation antigen CD86	942	**/**	**/**
	Q03167	Transforming growth factor beta receptor type 3	7049	**/**	**/**
	P50591-1	Tumor necrosis factor ligand superfamily member 10	8743	**/**	**/**
	P19438-1	Tumor necrosis factor receptor superfamily member 1A	7132	**/**	**/**
	P43489	Tumor necrosis factor receptor superfamily member 4	7293	Up	/
	P25942-1	Tumor necrosis factor receptor superfamily member 5	958	Up	/
	P25445-1	Tumor necrosis factor receptor superfamily member 6	355	/	/
	Q15628	Tumor necrosis factor receptor type 1-associated DEATH domain protein	8717	/	/
	P22749	Granulysin	10578	Up	/
	P12544-1	Granzyme A	3001	/	/
	P10144	Granzyme B	3002	/	/
	Q14761	Protein tyrosine phosphatase receptor type C-associated protein	5790	/	/
	P30455	HLA class I histocompatibility antigen, A-36 alpha chain	3105	Up	/
	P30479	HLA class I histocompatibility antigen, B-41 alpha chain	3106	Up	/
	P01889	HLA class I histocompatibility antigen, B-7 alpha chain	3106	Up	Up
	Q07000	HLA class I histocompatibility antigen, Cw-15 alpha chain	3107	Up	Up
	P30501	HLA class I histocompatibility antigen, Cw-2 alpha chain	3107	/	Up
	P01906	HLA class II histocompatibility antigen, DQ alpha 2 chain	3118	Up	/
	P79483	HLA class II histocompatibility antigen, DR beta 3 chain	3125	Up	Up
	Q30154	HLA class II histocompatibility antigen, DR beta 5 chain	3127	Up	Up
	Q95IE3	HLA class II histocompatibility antigen, DRB1-12 beta chain	3123	Up	/
	Q30134	HLA class II histocompatibility antigen, DRB1-8 beta chain	3123	Up	/
Complement	P13987	CD59 glycoprotein	966	/	/
	P00736	Complement C1r subcomponent	715	/	Up
	P06681-1	Complement C2	717	/	Up
	P01024	Complement C3	718	Up	Up
	P0C0L4-1	Complement C4-A	720	Up	Up
	P01031	Complement C5	727	Up	Up
	P13671	Complement component c6	729	/	Up
	P10643	Complement component C7	730	Up	Up
	P07357	Complement component C8 alpha chain	731	/	/
	P07358	Complement component C8 beta chain	732	/	/
	P02748	complement component C9	735	/	Up
	P08174-1	Complement decay-accelerating factor	1604	/	/
	P00751-1	Complement factor B	629	/	/
	P17927	Complement receptor type 1	1378	/	/
Coagulation	P01009-1	Alpha-1-antitrypsin	5265	/	/
	P08697-1	Alpha-2-antiplasmin	5345	/	Up
	P01008	Antithrombin-III	462	Up	Up
	P00740	Coagulation factor IX	2158	Up	Up
	P12259	Coagulation factor V	2153	Up	Up
	P00451	Coagulation factor VIII	2157	/	/
	P00742	Coagulation factor X	2159	Up	Up
	P00488	Coagulation factor XIII A chain	2162	/	/
	P05160	Coagulation factor XIII B chain	2165	Up	Up
	P02671-1	Fibrinogen alpha chain	2243	/	/
	P02675	Fibrinogen beta chain	2244	/	/
	P02679	Fibrinogen gamma chain	2266	/	/
	P05546	Heparin cofactor 2	3053	Up	Up
	P36955	Pigment epithelium-derived factor	5176	Up	Up
	P05155	Plasma protease C1 inhibitor	710	Up	Up
	P00734	Prothrombin	2147	Up	Up
	Q8IW75	Serpin A12	145264	/	/
	Q96P63	Serpin B12	89777	/	/
	P29508	Serpin B3	6317	/	/
	P50454	Serpin H1	871	/	/
Nanovesicle marker/Tetraspanin	P21926	CD9 antigen	928	/	Up
	P08962	CD63 antigen	967	/	/
	P60033	CD81 antigen	975	/	Up
	P27701	CD82 antigen	3732	Up	Up
Vesicle trafficking	Q99816	Tumor susceptibility gene 101 protein	7251	/	/
	Q15836	Vesicle-associated membrane protein 3	9341	/	/
	O95183	Vesicle-associated membrane protein 5	10791	/	/
	Q99698-1	Lysosomal-trafficking regulator	1130	/	/
	Q13571	Lysosomal-associated transmembrane protein 5	7805	/	/
	P11279	Lysosome-associated membrane glycoprotein 1	3916	/	/
	P13473-1	Lysosome-associated membrane glycoprotein 2	3920	/	/
	Q86Y82	Syntaxin-12	23673	/	/
	P32856	Syntaxin-2	2054	/	/
	O43752	Syntaxin-6	10228	/	/
	O15400	Syntaxin-7	8417	/	/

a *Up indicates proteins found overexpressed in NK-cell-derived microvesicle (NKMV) (abundance ratio NKMV/NKTotExtr > 1.5 and adjusted P < 0.05)*.

b *Up indicates proteins found overexpressed in NKExo (abundance ratio NKExo/NKTotExtr > 1.5 and adjusted P < 0.05)*.

### Effects of NKEVs on T Cells

**Figure 7:**
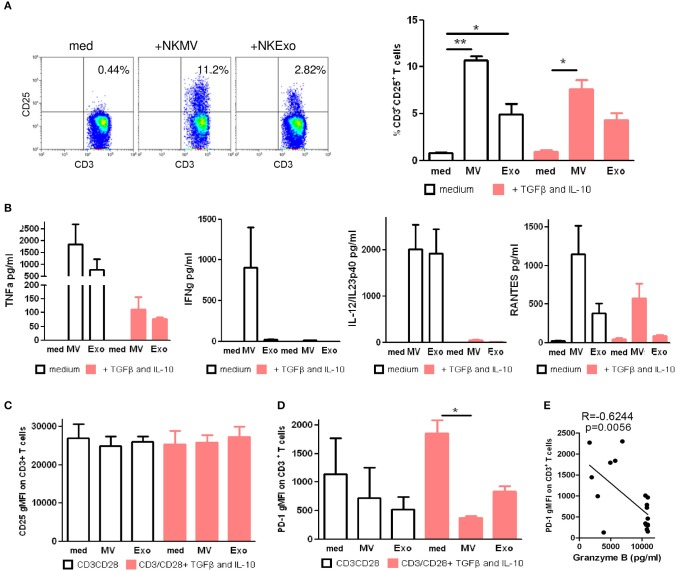
Effects of natural killer cell-derived extracellular vesicles (NKEVs) on T cells. **(A)** Flow cytometry analysis of CD25 expression by CD3^+^ gated T cells in peripheral blood mononuclear cells (PBMCs) evaluated after 72 h of culture with NK-cell-derived microvesicle (NKMV) and NKExo. Left panels: representative dot plots of a healthy donor. Right panel: the graph shows the results obtained with PBMCs of different healthy donors (*n* = 3), in the presence or absence of transforming growth factor beta (TGFβ)/interleukin (IL)-10 (10 ng/ml each). **(B)** Cytometric bead array (CBA)-measured cytokine production of 72 h PBMCs cultured as described in **(A)**. **(C)** Geo mean fluorescence intensity (gMFI) of CD25 expression of CD3^+^ gated T cells in CD3/CD28 activated PBMCs in the presence or absence of NKMV and NKExo and/or TGFβ/IL-10 (10 ng/ml each). **(D)** PD-1 gMFI on gated CD3^+^ T cells as in **(C)**. **(E)** Pearson correlation analysis of PD-1 gMFI and GRZB production. Results are shown for different healthy donors (*n* = 3). Statistical significance was achieved by paired *t*-test. Means ± SD are plotted, *p < 0.05; **p < 0.01.

To investigate the outcome of NKEVs and T-cell interactions, we isolated PBMCs from peripheral blood of healthy donors and incubated them for 72 h with NKMV (15 μg) or NKExo (30 μg) in the presence or absence of soluble IL-10 and TGFβ to mimic an immune suppressed environment. We could measure a statistically significant T-cell activation detectable as CD25 upregulation in CD3^+^ cells, which was more pronounced in the presence of NKMV than in the presence of NKExo (Figure 7A), confirming our hypothesis of an immune stimulatory role of NKEVs. NKEV-induced T-cell activation was still detectable in the presence of IL-10/TGFβ, even though at lower levels (Figure 7A), and was accompanied by a release of tumor necrosis factor alpha (TNFα), IFNγ, IL-12/IL-23p40, and RANTES (Figure 7B). The production of these cytokines was substantially reduced under the same conditions when IL-10/TGFβ cytokines were added, although even in this suppressive milieu, NKEVs induced the release of TNFα and RANTES by PBMCs (Figure 7B). In contrast, if we depleted CD14^+^ cells from PBMCs, the direct stimulatory effect of NKMV and NKExo in T cells was less evident, although we could measure a significant increase in the percentage of CD25^+^ CD4 T cells in the presence of NKMV (Figure S7A). These data suggest a role of monocytes in NKEV-mediated T-cell activation on the one hand, but that NKEVs may also influence T cells in their absence on the other hand. In the presence of CD3/CD28 stimulation and/or soluble IL-10 and TGFβ for 72 h, we could detect no differences in CD25 upregulation of CD3^+^-gated T cells in PBMCs, suggesting that NKMV or NKExo did not influence this process (Figure 7C). Similar results were obtained in the absence of CD14^+^ cells, although as for unstimulated CD4 T cells, a nonsignificant upregulation of CD25 expression by CD4 T cells was detectable in the presence of NKMV (Figure S7B). In PBMC experiments, we also concomitantly measured PD-1 expression on CD3/CD28 stimulated T cells and observed that the presence of NKMV and NKExo led to a decrease in fluorescence intensity of this immune checkpoint, an effect that was accentuated by adding TGFβ and IL-10 (Figure 7D). Cytokine detection in conditioned media of PBMCs collected at the end of stimulation showed remarkable amounts of granzyme B majorly deriving from NKEVs. Interestingly, statistical analysis revealed a significant inverse correlation between granzyme B and PD-1 geometric mean fluorescent intensity (gMFI) of T cells (Figure 7E). In summary, our results suggest that NKEVs may contribute to T-cell activation.

### Effects of NKEVs on Monocytes

**Figure 8:**
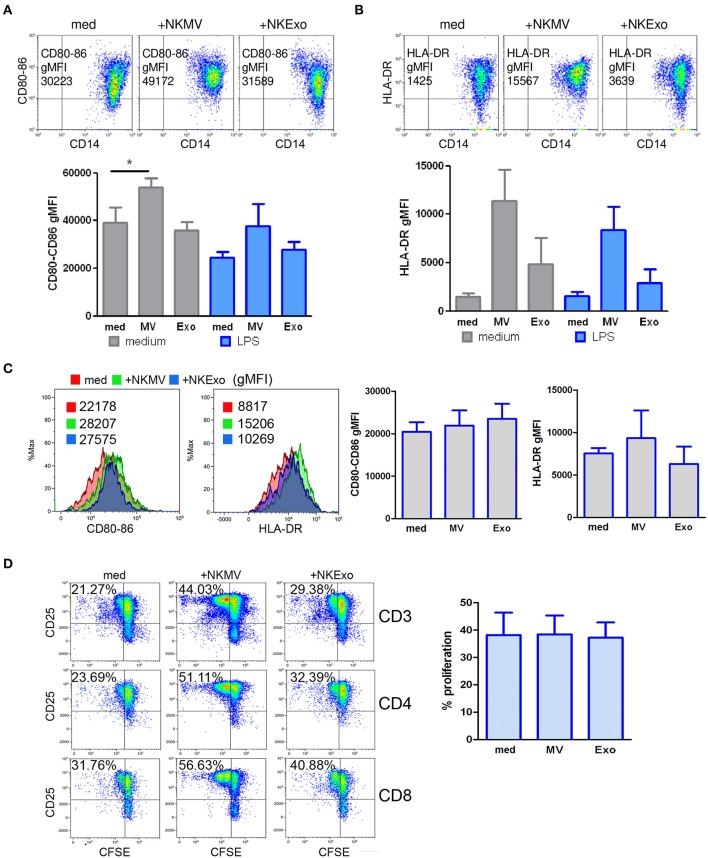
Effects of natural killer cell-derived extracellular vesicles (NKEVs) on monocytes. **(A)** Flow cytometry analysis of CD80–CD86 geo mean fluorescence intensity (gMFI) of gated CD14^+^ cells in peripheral blood mononuclear cells (PBMCs) cultured in the presence or absence of NK-cell-derived microvesicles (NKMV), NKExo, and/or lipopolysacharride (LPS) for 24 h. Upper panels: representative dot plots showing CD80–CD86 expression in the presence of NKMV and NKExo, lower panel: graphical summary of different healthy donors. **(B)** Flow cytometry of human leukocyte antigen DR isotype (HLA-DR) gMFI of CD14^+^ gated monocytes as in **(A)**. Results for different donors (*n* = 3) are shown. **(C)** Effects of NKEVs on isolated monocytes, measured by flow cytometry after 24 h culture of CD14^+^ cells with NKMV or NKExo. Left panels: gMFI of CD80-86 and HLA-DR expression by monocytes of one healthy donor. Right panel: results from different healthy donors (*n* = 3). **(D)** Stimulatory potential of monocytes preconditioned with NKMV and NKExo. Left panels: flow cytometry analysis of 72 h proliferation and CD25 expression by CD3, CD4, and CD8 T cells cultured in the presence of monocytes (medium), monocytes preconditioned with NKMV or NKExo. Right panels: graphical summary showing results from different healthy donors (*n* = 3). Percentage of proliferation is indicated. Statistical significance was achieved by paired *t* test. Means ± SD are plotted, *p < 0.05.

We studied the effects of NKEVs on monocytes in PBMCs in the presence or absence of 24 h LPS to recapitulate tolerogenic conditions (36). Additional experiments were performed on purified CD14^+^ cells to evaluate the direct effects of NKEVs. NKEV-conditioned monocytes were also tested for their stimulatory activity on autologous activated T cells. In PBMCs, we could assess a statistically significant increase in the costimulatory molecules CD80 and CD86 on gated CD14^+^ cells in the presence of NKMV. If LPS was present in the cultures, we observed a downregulation of CD80 and CD86, which was countered by NKMV (Figure 8A). These data suggest that NKMV possess stimulatory properties that lead to an upregulation of costimulatory molecules even if monocytes were exposed to tolerogenic LPS stimulation. Similar results were obtained for HLA-DR expression, where we could observe an induction mediated also by NKExo. Again, the activating potential of NKEVs was evident in the presence of LPS (Figure 8B). Compared to monocytes in PBMCs, purified CD14^+^ cells displayed less upregulation of the costimulatory molecules CD80-86 and HLA-DR if cultured with NKEVs. We could measure an increase in fluorescence intensity in monocytes only in one out of three healthy donors (Figure 8C). NKEV-conditioned monocytes were also tested for their stimulatory potential on autologous T cells in the presence of CD3/CD28 stimulation. Excess NKEVs were removed from monocytes before incubation with the CD14-depleted PBMCs. Results showed that, in one case out of three, NKEV-conditioned monocytes displayed an increased stimulatory activity on T-cell proliferation with respect to unconditioned ones, indicating that NKEVs can indeed positively influence activated T cells (Figure 8D).

### Effects of NKEVs on NK Cells

**Figure 9:**
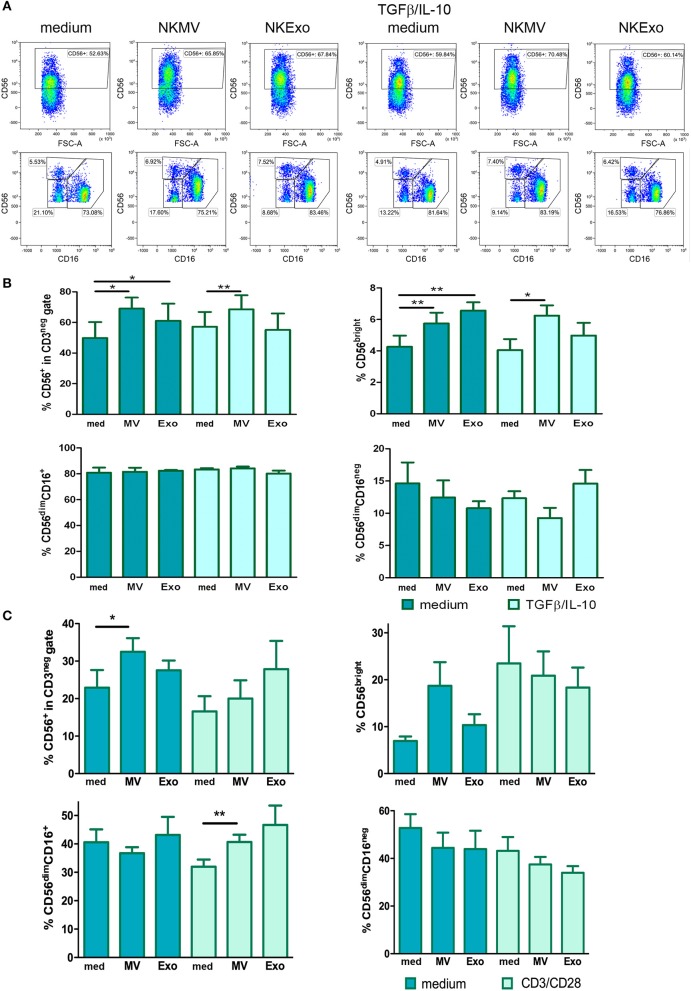
Effects of natural killer cell-derived extracellular vesicles (NKEVs) on NK cells. **(A)** Upper panels: representative dot plots showing CD56^+^ total NK cells in the CD3^negative^ lymphocyte gate after culturing PBMCs for 72 h in the presence or absence of NK-cell-derived microvesicle (NKMV) and NKExo and/or transforming growth factor beta (TGFβ)/interleukin (IL)-10 (10 ng/ml each). Lower panels: representative dot plots showing the percentage of NK cell subpopulations in the CD56^+^ gate for each culture condition as in upper panels: CD56^bright^, CD56^dim^CD16^+^, and CD56^dim^CD16^neg^ NK cells. **(B)** Graphs show the results obtained for total NK cells and each subpopulation shown in **(A)** for different healthy donors (*n* = 3). **(C)** Graphs show total NK cells and NK subpopulations after 72 h culture of the CD14^negative^ fraction in the presence or absence of NKMV or NKExo and/or CD3/CD28 stimulation. Results were obtained with cells from different donors (*n* = 3). Statistical significance was achieved by paired *t* test. Means ± SD are plotted, *p < 0.05; **p < 0.01.

The effects of NKEVs on NK cells were investigated in PBMCs from healthy donors cultured for 72 h in the presence or absence of TGFβ and IL-10. NK cells, gated as CD56^+^ in the CD3^neg^ gate (37), displayed an increase in percentage if NKEVs were present. This effect was particularly evident for the NKMV fraction, which maintained its stimulatory activity also in the TGFβ/IL-10 condition (Figure 9A, upper panels). Upon evaluation of the NK cell subpopulations, we could determine higher levels of CD56^bright^ cells, the IFNγ-producing population, also in the presence of TGFβ/IL-10. The cytotoxic CD56^dim^CD16^+^ cells increased only in the absence of TGFβ/IL-10, while CD56^dim^CD16^neg^ resting NK cells decreased (Figure 9A, lower panels). Differences were statistically significant in the case of total NK cells (Figure 9B, left upper panel) and CD56^bright^ NK cells (Figure 9B, right upper panel), where effects were mediated by both NKMV and NKExo. In the presence of TGFβ/IL-10, only effects mediated by NKMV maintained their statistical significance. No statistically significant differences could be assessed for CD56^dim^CD16^+^ and CD56^dim^CD16^neg^ NK cells (Figure 9B, lower panels). We also evaluated the activity of NKEVs on NK cells in PBMCs depleted from monocytes, in the presence or absence of CD3/CD28 T-cell stimuli. Similarly to what we observed in PBMCs, also in the absence of monocytes, NKMV led to a statistically significant increase in NK cells (Figure 9C, upper left panel). Interestingly, the presence of T-cell stimuli led to a significant increase in percentage of CD56^dim^ NK cytotoxic cell population concomitantly with a trend to decrease in CD56^dim^CD16^neg^ and CD56^bright^ NK cells (Figure 9C), an effect we could not detect if monocytes were present (data not shown).

### Circulating NK-Derived Exosomes and PBL/NK Cells in Healthy Donors and Melanoma Patients

**Figure 10:**
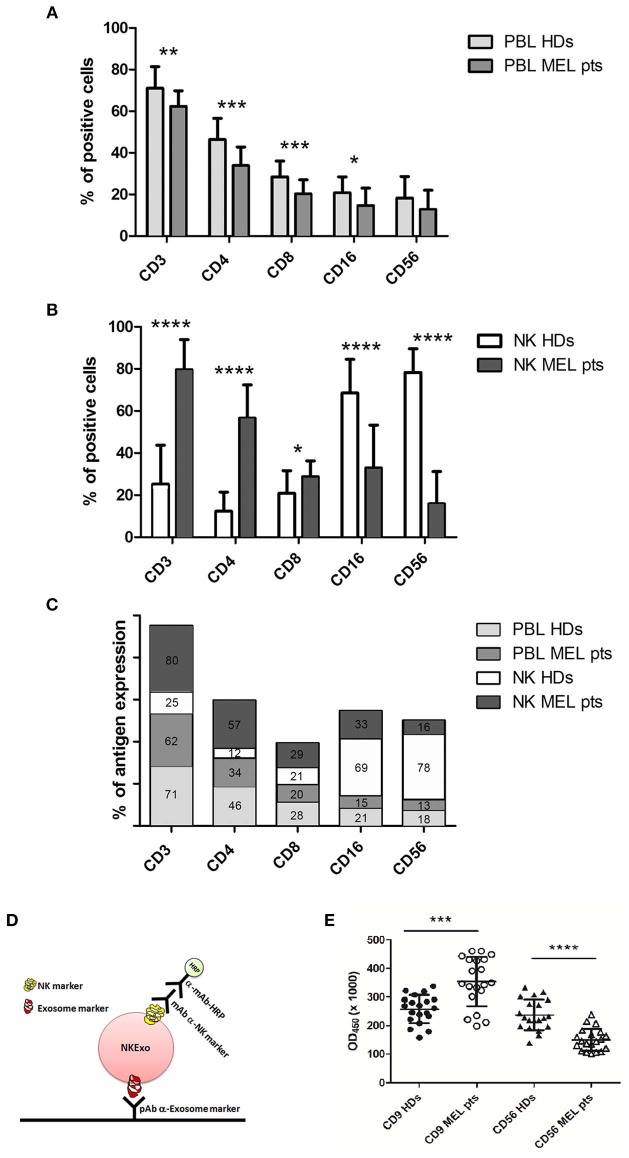
Characterization of circulating peripheral blood lymphocyte (PBL) and natural killer (NK) cells and quantification of plasma NKExo. **(A)** Immune phenotype analysis of freshly circulating PBL from healthy donor (HDs) and melanoma patients (MEL pts). **(B)** Immune phenotype analysis of 15 days-expanded NK cells from HDs and melanoma patients PBL. **(C)** Percent of indicated CD antigen expression in freshly PBL and 15 days expanded NK cells of HDs and melanoma patients. **(D)** Graphic scheme of “homemade” ELISA to detect and quantify NKExo in human plasma. (**(E)** Quantification of all circulating exosomes, by tsg-101 capture and CD9 detection and also of NKExo by tsg-101 capture and CD56 detection in plasma of HDs and melanoma patients, using the NKExoELISA test. Statistical significance was achieved by unpaired *t* test. Means ± SD are plotted, *p < 0.05; **p < 0.01; ***p < 0.001; ****p < 0.0001.

Several studies correlate cancer with NK-cell absence or dysfunction, highlighting the value of NK cells in cancer immunosurveillance. It has been demonstrated that lessened NK-cell-mediated cytotoxicity is often associated with increased risk of cancer development (5). With this premise, we first characterized freshly isolated peripheral blood lymphocyte (PBL) from melanoma patients and compared them with those from healthy donors (Figure 10A). The percentage of CD3, CD4, CD8, CD16, and CD56 cells in cancer patients' PBL was significantly decreased with respect to healthy donors, as shown in Figure 10A. Next, we evaluated the ability of NK cells to expand using the method previously reported (22). After 15 days, melanoma-patient-derived PBL cultures contained only few alive cells, and these expressed a different pattern of differentiation cluster compared to cells expanded from healthy donors' PBL (Figures 10B,C). In particular, healthy donor NK cells proliferated 30–40 times more and showed 80–95% of differentiation toward the NK phenotype, displaying higher expression of CD56 and CD16 molecules. On the opposite, melanoma patients' PBL neither proliferated nor differentiated into NK cells, expressing essentially CD3 and CD4 molecules (Figure 10C).

To test if this NK cell function deficiency could be detected in the liquid biopsy, we performed a homemade ELISA test (NKExoELISA) for the capture and relative quantification of NKEVs in human plasma. NKExoELISA detects NKExo in at least 1 ml of plasma and displays sensitive results, thus excluding MV. Probably, the major size of MV, compared to exosomes, prevented the antibody from recognizing and binding the antigen exposed on microvesicles. With the NKExoELISA, we capture intact circulating exosomes by a polyclonal antibody anti-tsg101, a typical exosome marker, and then detect exosomes released specifically by NK cells with a monoclonal anti-CD56 antibody (Figure 10D, graphical scheme). The analysis performed on the plasma of healthy donors compared to melanoma patients clearly showed that the amount of all circulating exosomes in melanoma patients was higher than in healthy donors (tsg-101^+^/CD9^+^ exosomes), while the fraction of tsg-101^+^/CD56^+^ exosomes (NKExo) was significantly lower (Figure 10E).

## Discussion

In view of a potential future application of NKEVs in support of cancer therapies, we studied EVs released into the extracellular milieu by NK cells from healthy donors to provide a photograph of the physiological content of these nanovesicles. We characterized NKEVs, both NKMV and NKExo, produced by human *ex vivo*-expanded healthy donor NK cells by NTA and electron microscopy and by LC-MS/MS mass spectrometry to get deeper insight into their morphology and protein composition. We also provided first evidence of their effects on immune cells in a healthy donor setting. Furthermore, with the intent of exploiting NKExo as surrogate of circulating NK cells, we conducted a homemade immunoenzymatic test to capture and quantify NKExo from human plasma. Our NKExoELISA is the first attempt to measure the human NK cell status via EVs and allowed us to quantify lower amounts of NKExo in the plasma of melanoma patients with respect to healthy donors, likely reflecting the biological defects of circulating NK cells in cancer patients.

We found that NK cells, in addition to exosomes, also release microvesicles. The size that we measured for NKExo, 124.8 ± 3.8 mean and 91.5 ± 6.7 mode, corresponds to the size defined for exosomes (30–150 nm), while NKMVs ranged from 315.2 ± 4.8 mean and 210.2 ± 7.1 mode, consistent with the one described for membrane-derived EVs (150–1,000 nm). Mass spectrometry showed that both NKExo and NKMV express specific biomarkers, high levels of cytoskeletal (actin, myosin, ERM proteins), cytosolic [14-3-3 protein, glyceraldehyde 3-phosphate dehydrogenase (GAPDH)], heat shock (e.g., HSP60, HSP70), antigen presentation (i.e., MHC-I, MHC-II), and plasma membrane proteins, i.e., the tetraspanins CD9, CD63, CD81, CD82, as well as proteins involved in vesicle trafficking, such as Tsg101 and several syntaxins. The expression of all these proteins on NKEVs is in accordance with the definition of EVs given by the International Society for Extracellular Vesicles (ISEV) (35, 38). Their protein composition postulates an involvement of NKEVs in cell adhesion, immune response, homing, cytotoxicity, coagulation, and complement cascade. NKMV and NKExo share several proteins, but with different degrees of significance, as in the case of proteins involved in complement activation, immune response, and proteolysis, resulting in more enrichment in NKExo than in NKMV. On the other side, the retinoid and lipoprotein metabolic processes resulted in significant enrichment in NKMV. NKEVs express several cell adhesion molecules (CAMs). The integrins facilitate cell–extracellular matrix adhesion and activate internal signaling transduction pathways (39). Integrin-mediated sensing, stiffening, and remodeling of the tumor stroma are key steps in cancer progression supporting invasion, acquisition of cancer stem cell characteristics, and drug resistance (40). The expression of several integrins by NKEVs could disturb the functionality of the integrins expressed by cancer cells, entering for example in competition with integrin ligands in the tumor microenvironment (41). Other important CAMs expressed by NKEVs are selectins, which are molecules mediating physiological responses such as inflammation, immunity, and hemostasis and involving in constitutive lymphocyte homing (42). Elevated levels of selectin ligands in carcinomas are associated with poor prognosis (43), and selectins can activate signaling cascades that regulate immune responses within a tumor microenvironment (44). Probably, the expression of these CAMs implies an involvement of NKEVs in the complex cellular crosstalk, both in physiological and pathological conditions. As expected, NKEVs carry a large variety of proteins involved in immune responses: CD276 (B7-H3), an immune checkpoint (45) expressed by NK cells that is involved in self-tolerance and costimulation of innate immunity by augmenting proinflammatory cytokine release of TNFα, IFNγ, IL-6, IL-1beta, and IL-12p70 from LPS-stimulated monocytes/macrophages; CD55 and CD59 complement regulators that could enable EVs to escape from complement attack, thus potentially prolonging their circulatory availability and stability (46); CD97, an activation-induced antigen on leukocytes and a critical mediator of host defense that binds CD55 and that is upregulated to promote adhesion and migration to sites of inflammation (47). It has been acknowledged that exosomes released by immune cells may act as antigen-presenting vesicles, stimulating antitumoral immune responses, or as inducers of tolerogenic effects suppressing inflammation (48). In fact, it has been demonstrated that although unstimulated NK cells do not express MHC class II, activated NK cells express HLA-DR and can initiate MHC class-II-dependent CD4^+^ T-cell proliferation (49, 50). Early studies showed that immune-cell-derived EVs carry MHC classes I and II (51). Surprisingly, our experiments show that also NKEVs express MHC classes I and II. These data suggest that NKEVs could have a role not only in MHC class I self-recognition but also in antigen presentation. NKEVs express multiple cytotoxic proteins, such as perforin, FasL, granzyme A and B, granulysin (22, 25), as well as molecules that activate NK-mediated cytolysis, i.e., CD40L. Indeed, reports indicate that NKEVs can trigger multiple killing mechanisms (52). NK cells and probably also NKEVs may possess regulatory functions (6). By means of IFNγ and TNF, NK cells can promote the maturation of DCs, which in turn activate NK cells via IL-12. We report in this study that NKEVs also express several TNF receptors and ligands and carry different types of interferon-induced transmembrane proteins (IFITM). Therefore, in a specular way to the NK cells, the NKEVs could influence adaptive immune responses by directly acting on T and B cells. In the inflamed lymph node, NK cells promote the priming of CD4^+^ T helper type 1 cells by secreting IFNγ (53), a phenomenon that could me mediated also by NKEVs. In addition, NKEVs carry different interferon regulatory factors (IRFs). Regarding coagulation-related proteins, this is the first report showing their expression by NKEVs and with regulatory functions, i.e., factors V, VIII, IX, X, and XIII, fibrinogen, prothrombin, anticoagulant antithrombin, protein S, and protein C. In cancer, coagulation facilitates tumor progression through the release of platelet granule contents, inhibition of NK cells, and recruitment of macrophages (54–56). Plasma exosomes in epithelial ovarian cancer seem to cover a potential role in the coagulation cascade (57), and treatment of cancer patients with anticoagulant drugs led to diminished metastasis (56). Thus, NKEVs could contribute to coagulation homeostasis and cancer surveillance.

Given that NKEVs contained numerous proteins involved in immune regulation, we were not surprised to detect modulatory effects on different immune cells. Our experiments were performed with healthy donors' PBMCs cultured with NKEVs derived from healthy donors' NK cells to assess the physiological properties of these vesicles. In this setting, we also included conditions aimed at recapitulating an immune suppressive state. Overall, our results show that NKEVs exhibit stimulatory activities on monocytes, T cells, and NK cells in a PBMC context and to a lesser extent also on isolated monocytes and on T and NK cells in the CD14-depleted fraction, potentially suggesting that a direct and indirect stimulation by NKEVs, which could occur simultaneously in a PBMC setting, may represent an optimal condition for stimulation by NK-cell-derived EVs. Results of PBMC studies showed that NKEVs induced the expression of HLA-DR and costimulatory molecules on monocytes albeit the presence of LPS, a condition aimed at mimicking a tolerogenic environment (36). Again, in a PBMC setting, NKEVs stimulated the upregulation of CD25 by T cells, while in the presence of CD3/CD28 triggering they induced a downregulation of PD-1 in gated T cells that correlated with the presence of granzyme B, this latter deriving mostly from NKEVs but potentially also from CD3/CD28-stimulated cells. Although attenuated, these effects were still detectable in PBMC-NKEV cocultures containing TGFβ and IL-10, a condition we included to recapitulate an immune compromised microenvironment, such as in cancer (58). The NKEV-mediated stimulatory activity on the NK cell population was detectable in PBMCs as well as in CD14-depleted PBMCs, showing an increase in percentage of CD56^+^ total NK cells and the CD56^bright^ and CD56^dim^ subpopulations. NKEVs could engage with NK cells and amplify their inflammatory response, as reported for monocyte–NK cell interaction (59). Most of the observed effects were primarily mediated by the NKMV fraction, although also NKExo displayed stimulatory properties. This could depend on the higher content of stimulatory immune factors such as IL-2 and IFNγ, which we measured in NKMV with respect to NKExo. These soluble factors were detected by cytokine bead array in 15 μg NKMV and 30 μg NKExo protein after isolating them from the same volume of supernatant. This kind of measurement did not take into account the differences in vesicle size or concentration, thus suggesting that in half the protein amount, the MV carry more of some of the measured cytokines with respect to Exo. The increased stimulatory potential of MV might also depend on the bigger dimension of these EVs, which could facilitate a stronger EV–cell contact via enhanced available interaction surface. Thanks to their rich repertoire of adhesion molecules, NKEVs may bind to target cells to deliver their activating signals via surface–surface interaction directly. In fact, we hypothesize this to represent the main type of interaction with T and NK cells. In contrast, the interaction of monocytes with NKEVs may also be conditioned by internalization of the vesicles, potentially leading to a different response by monocytes as compared to surface–surface interaction. In addition, the differences in donor cells' response that we observed upon conditioning isolated monocytes with NKEVs and their consequent stimulatory potential on T-cell proliferation may suggest interdonor variability. In the absence of CD3/CD28 stimuli, NKMV induced the activation of CD4 T cells, a phenomenon that could rely on HLA-DR expressed by NKEVs, as previously shown for NK cells (50). The observed immunomodulatory effects still need further investigation; nonetheless, our results may represent a starting point for future evaluation of NKEVs stimulatory potential also on cancer-patient-derived immune cells, including myeloid-derived suppressor cells and regulatory T cells, major contributors to the detrimental phenomenon of immune suppression in cancer (60). Here, the activity of NKEVs could potentially contribute reversing immune suppression.

We performed a homemade ELISA test (NKExoELISA) for the capture and quantification of NK exosomes derived from human plasma of healthy donors and cancer patients. We previously developed a similar assay to detect and quantify plasma exosomes expressing Rab5B/CD63 or Rab5B/caveolin plasma exosomes and demonstrated that CD63^+^ exosomes or caveolin-1^+^ exosomes were significantly increased in melanoma and prostate cancer patients as compared to healthy donors (61, 62). Here, we performed a homemade ELISA that is able to capture specifically exosomes derived from NK cells. By NKExoELISA, we demonstrated that in melanoma patients, the amount of circulating NKExo is lower than in healthy donors. This last result is correlated with a low proliferation and no differentiation toward the NK phenotype of circulating PBLs of melanoma patients if subjected to the same *in vitro* NK expansion protocol as for healthy donor PBL (22).

Taken together our data suggest that NKExo and NKMV could cover a promising role in the support of NK-mediated immunosurveillance, potentially sustaining therefore a wide range of disease therapies. In particular, the use of NKEVs in combination with NK cells or immune checkpoint-based therapies may contribute to improving cancer treatment.

## Data Availability Statement

The raw data supporting the conclusions of this article will be made available by the authors, without undue reservation, to any qualified researcher.

## Ethics Statement

The studies involving human participants were reviewed and approved by the Ethical Committee of Azienda Policlinico Umberto I, University Sapienza, Rome, Italy. The patients/participants provided their written informed consent to participate in this study.

## Author Contributions

LL and VH were responsible for the study design, wrote and critically reviewed the manuscript, and supervised the study. CF and ES wrote the manuscript, provided technical support for exosome isolation and characterization, and performed many experiments. SCe contributed figures and tables and with EI expanded and characterized NK cells and PBL and also contributed to the discussion of mass spectrometry results. SCam and MC provided results of mass spectrometry and related figures. CC, SF, AC, and PS performed immunomodulation experiments. GP and SCal provided the plasma of melanoma patients and patient informed consent. LB and FI provided results of transmission and scanning electron microscopy and related images.

### Conflict of Interest

The authors declare that the research was conducted in the absence of any commercial or financial relationships that could be construed as a potential conflict of interest.
