# Supplementary figures and images for: Natural-Killer-Derived Extracellular Vesicles: Immune Sensors and Interactors
Source: Front Immunol. 2020 Mar 13;11:262. doi: 10.3389/fimmu.2020.00262 (PMC7082405; doi:10.3389/fimmu.2020.00262)

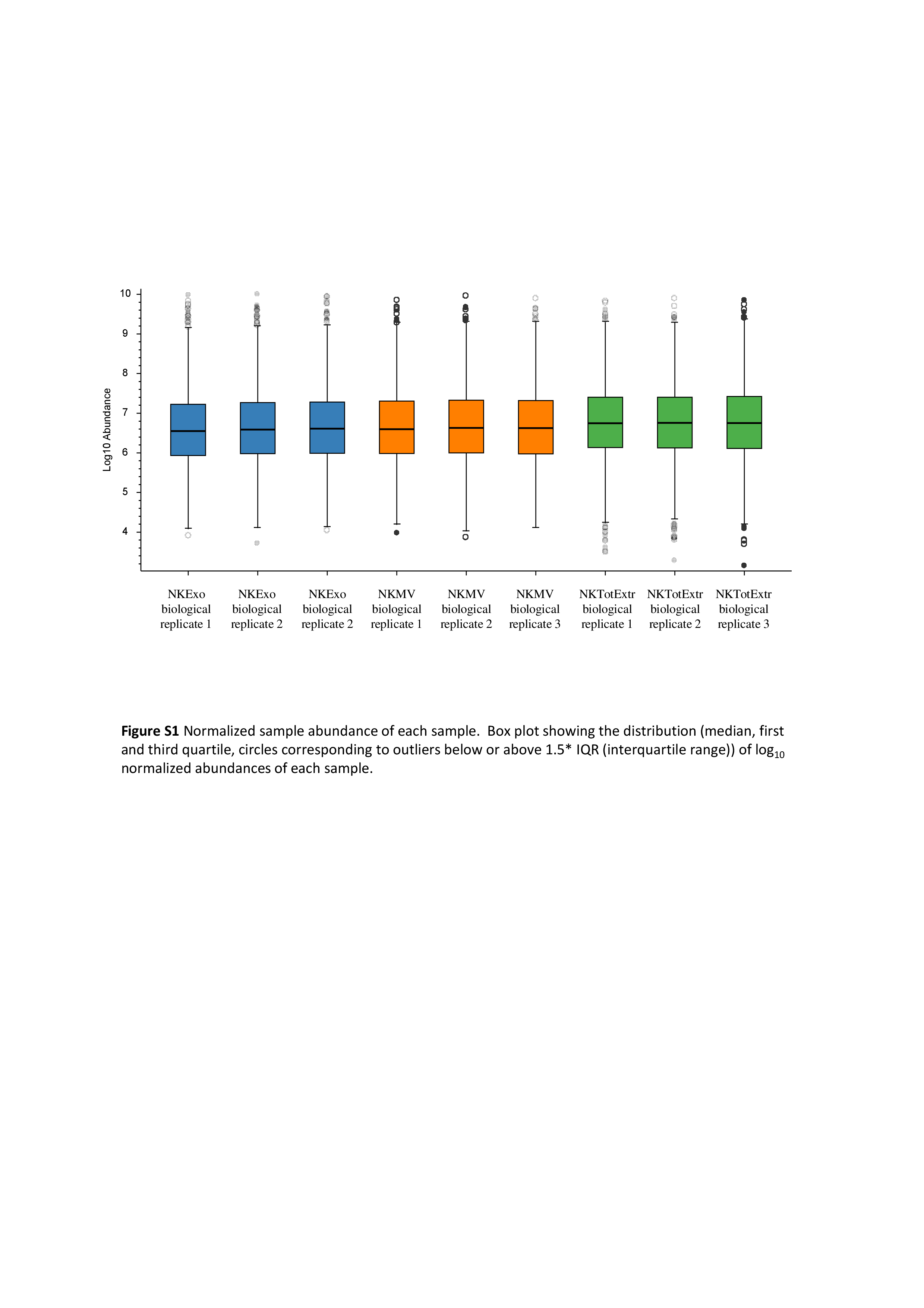

Supplement: Supplementary file 3 [file Image_1.TIF]

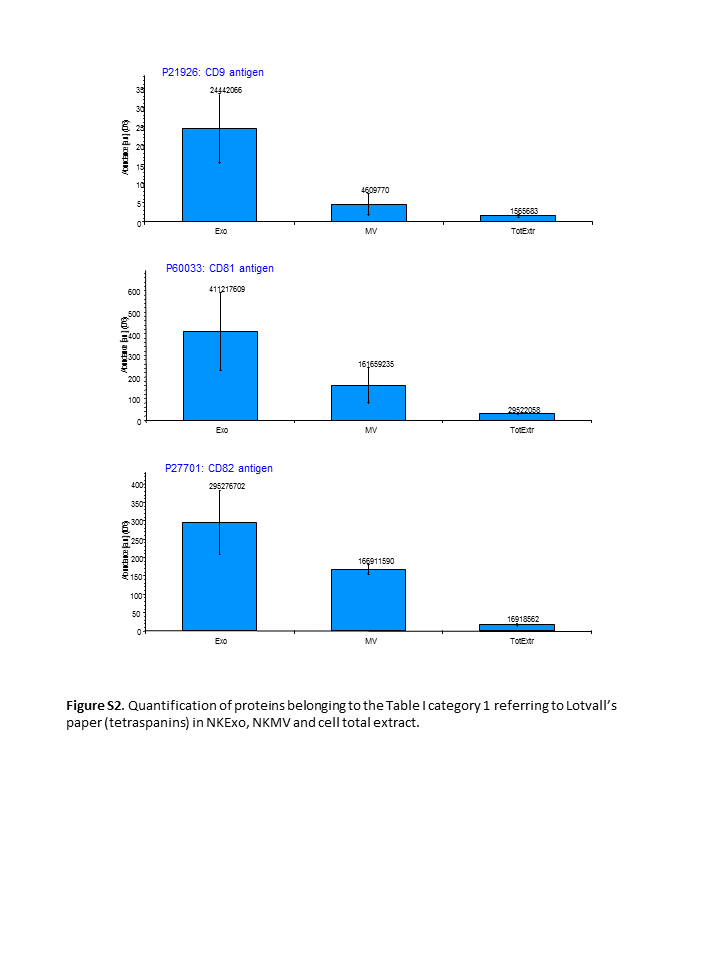

Supplement: Supplementary file 4 [file Image_2.TIF]

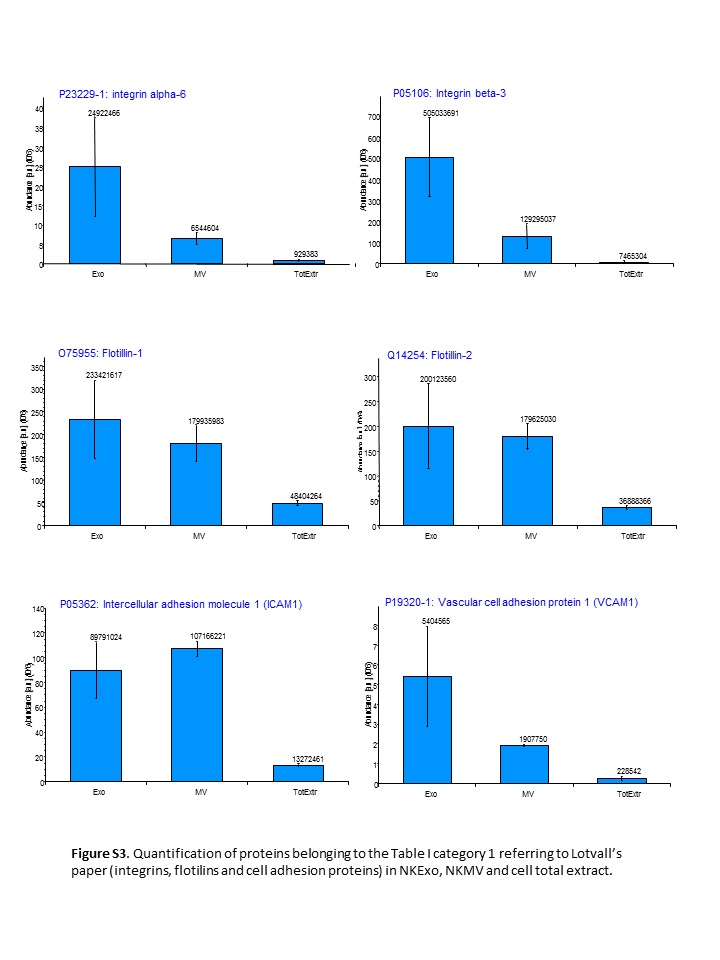

Supplement: Supplementary file 5 [file Image_3.TIF]

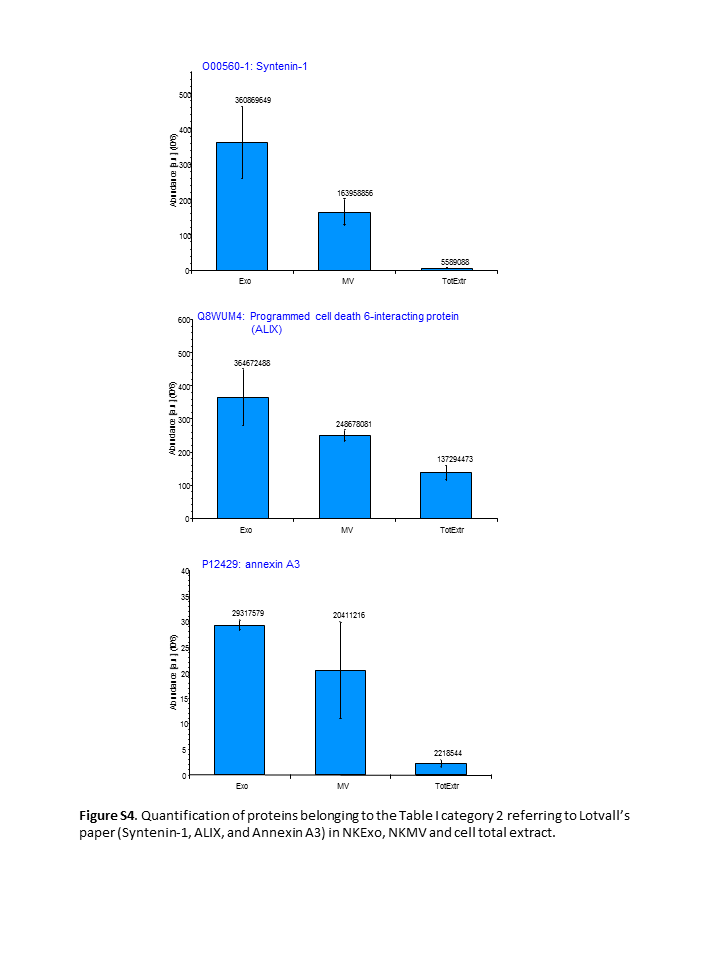

Supplement: Supplementary file 6 [file Image_4.TIF]

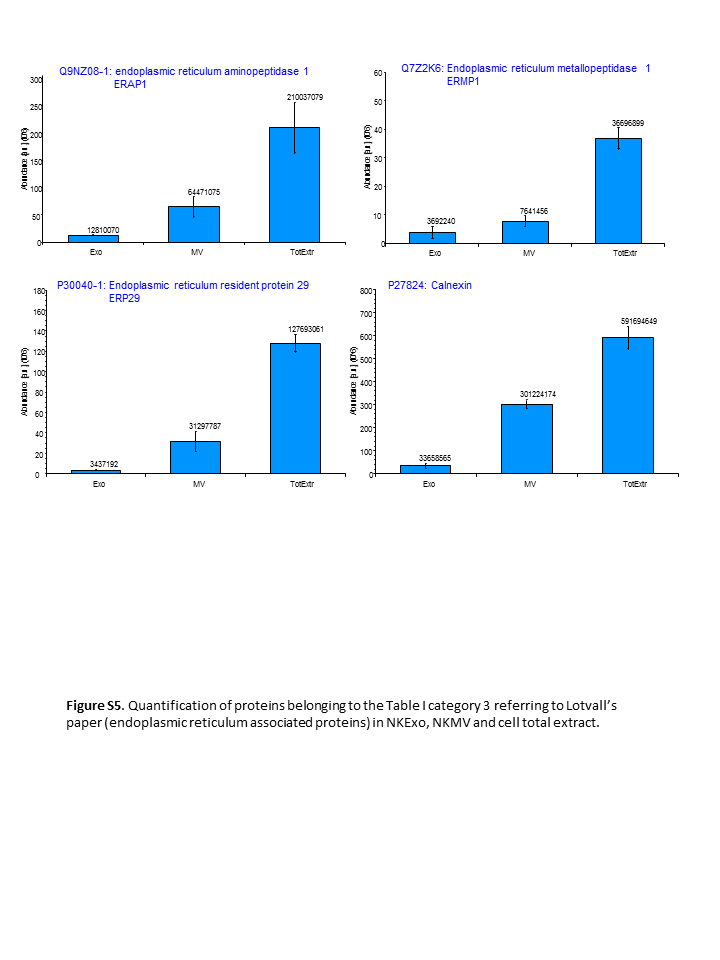

Supplement: Supplementary file 7 [file Image_5.TIF]

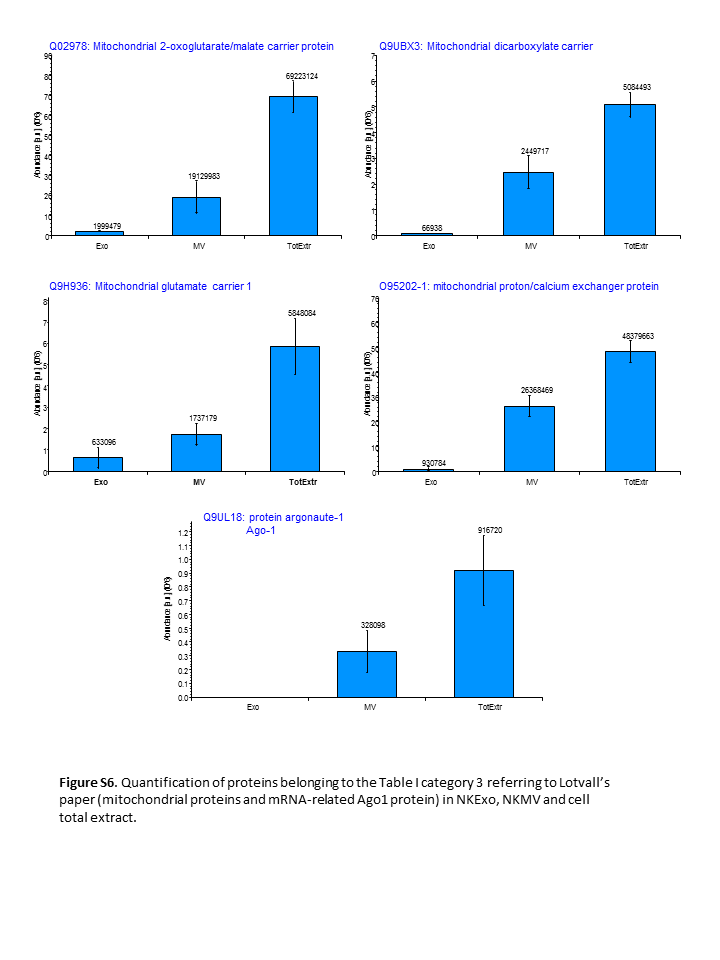

Supplement: Supplementary file 8 [file Image_6.TIF]

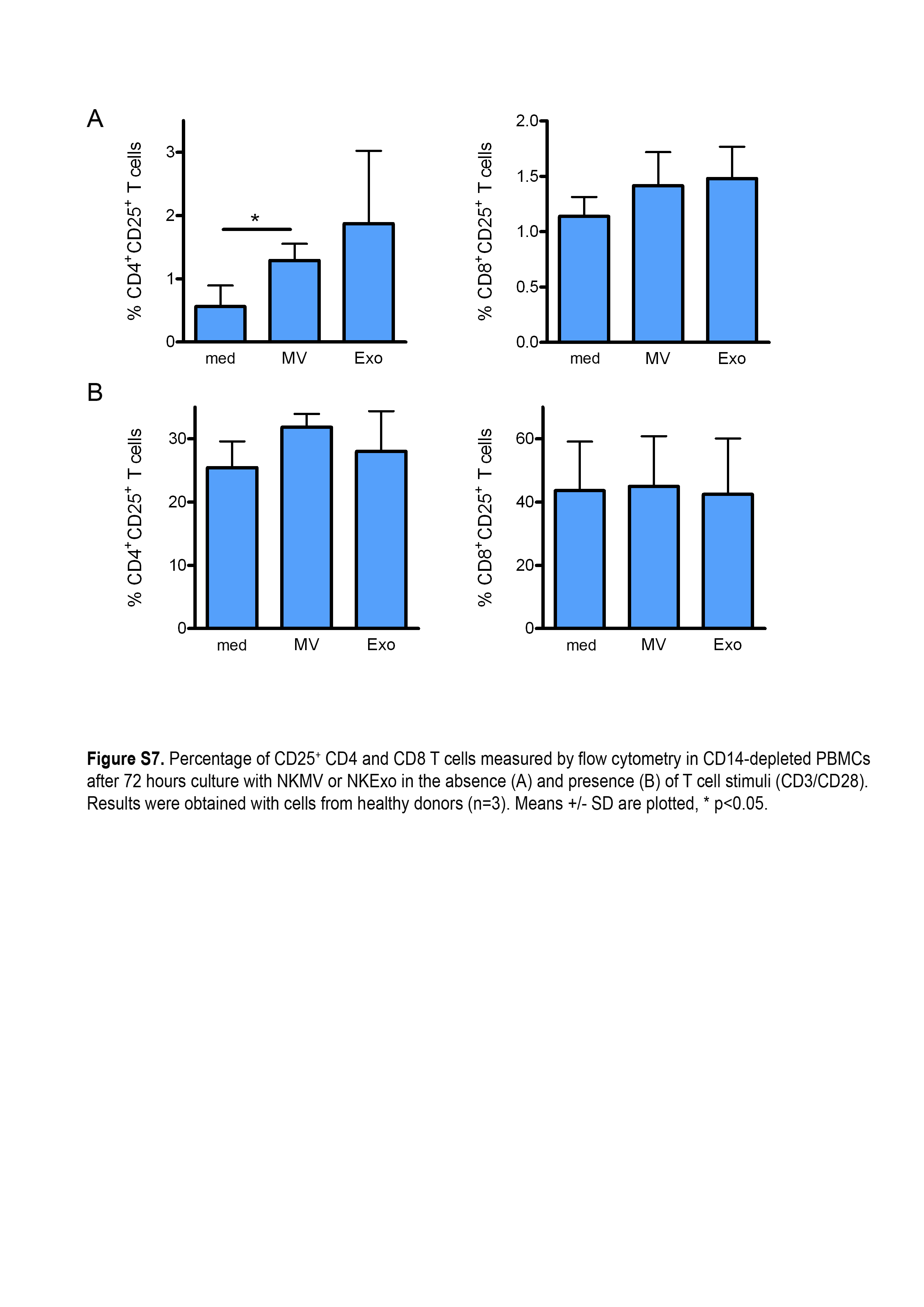

Supplement: Supplementary file 9 [file Image_7.TIF]
